# Longitudinal Assessment of Prenatal, Perinatal, and Early-Life Aflatoxin B_1_ Exposure in 828 Mother–Child Dyads from Bangladesh and Malawi

**DOI:** 10.1093/cdn/nzab153

**Published:** 2022-01-07

**Authors:** Joshua W Smith, Andrew J Matchado, Lee S-F Wu, Charles D Arnold, Sean M Burke, Kenneth M Maleta, Per Ashorn, Christine P Stewart, Saijuddin Shaikh, Hasmot Ali, Alain B Labrique, Keith P West, Parul Christian, Kathryn G Dewey, John D Groopman, Kerry J Schulze

**Affiliations:** Department of Environmental Health and Engineering, Bloomberg School of Public Health, Johns Hopkins University, Baltimore, MD, USA; Department of Nutrition and Institute for Global Nutrition, University of California, Davis, Davis, CA, USA; School of Public Health and Family Medicine, College of Medicine, University of Malawi, Blantyre, Malawi; Center for Human Nutrition, Department of International Health, Bloomberg School of Public Health, Johns Hopkins University, Baltimore, MD, USA; Department of Nutrition and Institute for Global Nutrition, University of California, Davis, Davis, CA, USA; Department of Environmental Health and Engineering, Bloomberg School of Public Health, Johns Hopkins University, Baltimore, MD, USA; School of Public Health and Family Medicine, College of Medicine, University of Malawi, Blantyre, Malawi; Tampere University, Faculty of Medicine and Health Technology, Center for Child, Adolescent and Maternal Health Research and Tampere University Hospital, Department of Pediatrics, Tampere, Finland; Department of Nutrition and Institute for Global Nutrition, University of California, Davis, Davis, CA, USA; Center for Human Nutrition, Department of International Health, Bloomberg School of Public Health, Johns Hopkins University, Baltimore, MD, USA; The JiVitA Project of Johns Hopkins University, Bangladesh, Gaibandha, Bangladesh; Center for Human Nutrition, Department of International Health, Bloomberg School of Public Health, Johns Hopkins University, Baltimore, MD, USA; The JiVitA Project of Johns Hopkins University, Bangladesh, Gaibandha, Bangladesh; Center for Human Nutrition, Department of International Health, Bloomberg School of Public Health, Johns Hopkins University, Baltimore, MD, USA; Center for Human Nutrition, Department of International Health, Bloomberg School of Public Health, Johns Hopkins University, Baltimore, MD, USA; Center for Human Nutrition, Department of International Health, Bloomberg School of Public Health, Johns Hopkins University, Baltimore, MD, USA; Department of Nutrition and Institute for Global Nutrition, University of California, Davis, Davis, CA, USA; Department of Environmental Health and Engineering, Bloomberg School of Public Health, Johns Hopkins University, Baltimore, MD, USA; Center for Human Nutrition, Department of International Health, Bloomberg School of Public Health, Johns Hopkins University, Baltimore, MD, USA

**Keywords:** aflatoxin, mass spectrometry, diet, pregnancy, breastfeeding, infancy, cord blood, seasonality, toxicology

## Abstract

**Background:**

In utero or early-life exposure to aflatoxin, which contaminates staple crops in disadvantaged settings, may compromise pregnancy and infant outcomes, but investigations into the extent, persistence, and determinants of aflatoxin exposure at these life stages have lacked longitudinal data collection and broad geographic representation.

**Objectives:**

Aflatoxin exposure and selected determinants thereof were characterized in mother–child dyads with serial plasma/serum samples in prenatal, perinatal, and early life in Malawi and Bangladesh.

**Methods:**

Circulating aflatoxin B_1_ (AFB_1_)–lysine albumin adducts were measured in dyads from Bangladesh (*n *= 573; maternal first and third trimester, 3 mo postpartum, cord blood, infant 24 mo) and Malawi (*n *= 255; maternal second and third trimester, 6 mo postpartum, infant 6 and 18 mo) with isotope dilution mass spectrometry. We examined AFB_1_-lysine adduct magnitude, persistence, seasonality, and associations with infant feeding, and estimated daily AFB_1_ intake.

**Results:**

Maternal AFB_1_-lysine was higher in Malawi (98% detectable; median: 0.469, IQR: 0.225–1.027 pg/µL) than in Bangladesh (59%; 0.030, nondetectable [nd]–0.077 pg/µL). Although estimated dietary exposure in Malawi was temporally stable (648 ng AFB_1_/day), estimated intake in Bangladesh was reduced by 94% between rainy and winter seasons (98 to 6 ng/day). AFB_1_-lysine was low in cord blood from Bangladesh (15% detectable; 0.045, 0.031–0.088 pg/µL among detectable) and in Malawian infants at 6 mo of age (0.072, nd–0.236 pg/µL), but reached maternal concentrations by 18 or 24 mo (Bangladesh: 0.034, nd–0.063 pg/µL; Malawi: 0.370, 0.195–0.964 pg/µL). In Malawian infants, exclusive breastfeeding at 3 mo was associated with 58% lower AFB_1_-lysine concentrations at 6 mo compared with other feeding modes (*P *= 0.010).

**Conclusions:**

Among pregnant women, aflatoxin exposure was persistently high in Malawi, while lower and seasonal in Bangladesh. Infants were partially protected from exposure in utero and with exclusive breastfeeding, but exposures reached adult levels by 18–24 mo of age. The Bangladesh and Malawi trials are registered at clinicaltrials.gov as NCT00860470 and NCT01239693.

## Teaser

Aflatoxin B_1_ exposure in rural Malawian and Bangladeshi mother–infant dyads differed in magnitude and seasonality, was reduced in utero and in infancy, but was akin to maternal exposure by 18 months of age.

## Introduction

Aflatoxins are mycotoxins produced by certain species of fungi that contaminate staple foods and proliferate in hot, humid environments, where crops may languish in fields or in poor storage facilities—conditions especially common in low- and middle-income countries (1–4). Aflatoxin contamination has been associated with specific staple crops, including maize, groundnuts, wheat, sorghum, and rice, but may occur in a variety of other foods as well (5). Levels of contamination can vary widely, resulting in human exposures that can range from nanograms to milligrams per day, depending upon the grain and amount consumed (6). Variation in dietary patterns and the wide heterogeneity in aflatoxin contamination in grains make it difficult to estimate human exposure using dietary questionnaires and spot testing of staple foods, thus complicating exposure surveillance and primary prevention efforts. Moreover, just as the bioavailability of nutrients affects their absorption in the gut, only a fraction of an aflatoxin exposure is absorbed and reflected in the internal dose (the amount of aflatoxin that enters the body and may exert downstream biological effects). This variability cannot be captured by questionnaire or food survey exposure assessment approaches.

The development and validation of quantitative techniques for the measurement of biomarkers of aflatoxin internal dose in humans have been critical for their application to etiologic, intervention, and prevention studies in high-risk populations across the globe. Since the chemical characterization of the aflatoxin B_1_-serum albumin lysine adduct (AFB_1_-lysine) (7), a number of methods have been deployed to determine these adduct concentrations in humans, with the current gold-standard method of measuring aflatoxin internal dose being the measurement of serum AFB_1_-lysine concentrations using isotope dilution mass spectrometry (IDMS) (8). The AFB_1_-lysine adduct is particularly valuable, since the half-life of human serum albumin in circulation [∼17 days (9, 10)] allows a single AFB_1_-lysine measurement to reflect a smoothed estimate of chronic aflatoxin exposure over the previous 1 to 3 mo. Regardless of the approach used for biomonitoring, however, a finding of significant aflatoxin exposure in a population not only raises concerns of direct effects of aflatoxin toxicity and carcinogenicity but, in developing countries, often coincides with limited dietary diversity and micronutrient deficiencies. Thus, when aflatoxin exposure is determined to be substantial, these findings should be viewed as a sentinel for poor nutritional status and low quality of staple grains (11).

While much of the international focus on aflatoxin contamination has been framed within its carcinogenic properties (3, 6), many of its other toxic effects can result in significant short- and long-term health consequences. In South Asia, Africa, and Central America, where both maternal and child health statuses are tremendously compromised, potential effects of aflatoxin exposure may be additionally manifested in noncarcinogenic endpoints (12, 13). Aside from its well-known role in hepatic carcinogenesis, aflatoxin induces many adverse local and systemic effects that impair normal organ and tissue function, resulting in inflammation, immune suppression, and growth retardation, all of which contribute to poor health (2, 3, 14). A role for aflatoxin in impairing reproductive outcomes and attenuating growth in childhood has been postulated primarily based on the experimental literature regarding aflatoxin exposure in poultry and livestock (4), but is additionally supported by observational cross-sectional reports and a limited number of longitudinal studies in humans. Several recent systematic reviews have focused on the available human studies (including a handful of randomized clinical trials) and aflatoxin biomarker measurements have been associated with adverse health outcomes during early life in some at-risk settings (14–19).

While prior studies have demonstrated the impact of seasonality on aflatoxin exposure and the potentially critical role of breastfeeding in mitigating exposure early in life, without longitudinal monitoring throughout pregnancy and early infancy (≤6 mo of age), potentially important implications of timing of exposure may be obscured. Additionally, while many studies examining the relation between aflatoxin exposure and child growth have been conducted in western sub-Saharan Africa, where groundnut and maize are predominant foods in the diet, fewer studies have investigated these questions in south or southeastern Asia, where stunting is common and often severe (20, 21), but which features substantially different dietary patterns and staple foods, including rice.

Thus, the objective of the present study was to quantify longitudinal aflatoxin exposure in over 800 mother–child dyads across prenatal, perinatal, and early life, at population research sites in eastern sub-Saharan Africa (Malawi) and southeast Asia (Bangladesh). These settings have broadly different dietary patterns, but common features include nutritional deficiencies among women and risk for poor growth and development among infants. Harnessing the strength of side-by-side analysis of samples collected within 2 community-based, randomized controlled trials of nutritional interventions and using the gold-standard IDMS method of AFB_1_-lysine biomonitoring, we ascertained the prevalence, magnitude, and timing of aflatoxin exposure in relation to seasonality and breastfeeding practices. We also provide estimates of dietary aflatoxin intake at the population level across both settings.

## Methods

### Subjects and study designs

This study utilized serum or plasma samples and data previously collected from 2 completed trials: the cluster-randomized, controlled, double-blind JiVitA-3 trial in the Gaibandha and Rangpur Districts of Bangladesh (clinicaltrials.gov registration NCT00860470) (22) and the individually randomized, controlled, partially double-blind iLiNS-DYAD-M trial in the Mangochi District of Malawi (clinicaltrials.gov registration NCT01239693) (23). The JiVitA-3 trial was approved by institutional review boards at the Johns Hopkins Bloomberg School of Public Health (Baltimore, MD, USA) and the Bangladesh Medical Research Council (Dhaka, Bangladesh). The iLiNS-DYAD-M trial was approved by the College of Medicine Research and Ethics Committee of the University of Malawi (Blantyre, Malawi) and the Ethics Committee of the Pirkanmaa Hospital District (Tampere, Finland). All participants confirmed their informed consent orally (JiVitA-3) or with signatures or thumbprints on consent forms (iLiNS-DYAD-M). In both studies, consent was provided with the understanding that their samples or (anonymized) data may be used in future analysis. Local communities were given opportunities to provide input on study design and implementation. All data used in the present analysis were anonymized; only principal investigators and appropriate members of the respective research teams have access to the secured, nonanonymized data.

Among many other endpoints, both studies collected data for the assessment of pregnancy outcomes and child anthropometrics from birth until 24 mo of age in the JiVitA-3 trial and until 18 mo of age in iLiNS-DYAD-M. The JiVitA-3 trial was designed to test the effects of an antenatal multiple micronutrient supplement (MMS) compared with a standard-of-care control iron and folic acid (IFA) supplement. Similarly, the iLiNS-DYAD-M trial also examined the effects of MMS and IFA prenatal supplementation (although with slightly different formulations than JiVitA-3), but used these controls as active comparators against an experimental prenatal lipid-based nutrient supplement (LNS) arm. Details on the formulations of these supplements have been published elsewhere (22, 23).

Newly married women aged 12 to 45 y old were recruited to the JiVitA-3 trial through a pregnancy surveillance program to identify and enroll women during the first trimester of pregnancy. A substudy in a predetermined geographic subregion of the JiVitA-3 trial site was conducted from June 2008 to February 2011 for more intensive data collection, including blood samples in early pregnancy (∼10 wk gestation; here designated M-1TM) and late pregnancy (32 wk gestation; M-3TM), and at 3 mo postpartum in women (M-3mo), in cord blood (C-Cord) in a limited subset of substudy participants, and in children at 2 y of age (C-24mo) in an even smaller subset. Among these available samples, a cohort of 1526 women was identified in whom complete serial data collection was available either through the cord blood study or through 3 mo postpartum; this cohort has been previously described (24), as has the group from which cord blood was collected (25, 26), and forms the sampling frame for this study.

Pregnant Malawian women seeking prenatal care at clinics within the iLiNS-DYAD-M study catchment area were recruited for enrollment, provided they were >15 y of age and ≤20 wk of gestation. A total of 869 women were enrolled for complete follow-up in the study and followed until their children were 18 mo of age; recruitment and follow-up occurred from October 2011 to April 2015. Blood samples were collected from mothers at baseline during the second trimester (M-2TM), 36 wk of gestation (M-3TM), and 6 mo postpartum (M-6mo). Blood samples were also collected from children 6 mo after birth (C-6mo) and at 18 months of age (C-18mo).

In both studies, extensive demographic, socioeconomic, dietary, anthropometric, and other data were collected to describe household, maternal, and infant characteristics. Infant feeding practices were assessed differently between sites, with the JiVitA project administering a questionnaire at 3 and 6 mo of age regarding feeding practices occurring in the prior ∼3 mo, to ascertain usual feeding practices as exclusive, predominant, or partial breastfeeding. The ILiNS-DYAD-M study used the Infant and Young Child Feeding Indicators questionnaire (27) at 3 and 6 mo of age to assess feeding practices in the last 24 h. The JiVitA-3 trial used the Food Access Survey Tool (FAST) (28) to determine household food security for the period encompassing the first 6 mo postpartum, while the iLiNS-DYAD-M study used the Household Food Insecurity Access Scale (HFIAS) (29) at maternal baseline enrollment. The Cronbach's ɑ value for the HFIAS instrument within the iLiNS-DYAD-M trial was 0.81, while the corresponding value for the FAST questionnaire was 0.85 in the JiVitA-3 trial.

### Sample selection and representation

**Supplemental Figure 1** depicts flow diagrams of sample selection for the current study, for each trial site. Aliquots from 1152 plasma samples from 230 mother–child dyads were selected for analysis from the iLiNS-DYAD-M trial. All of these 230 dyads had “complete” sets of plasma samples—that is, plasma was available for each sample type within a dyad (M-2TM, M-3TM, M-6mo, C-6mo, C-18mo). Plasma was also available for analysis at midpregnancy (M-2TM) and late pregnancy (M-3TM) from an additional 25 mothers (without matched postpartum or child samples), bringing the total number of pregnancies included to *n *= 255 and the total samples analyzed to *n *= 1202.

Based on available samples and outcome data, serum samples from JiVitA-3 pregnancies were selected for analysis in groups composed of the following combinations of maternal–child sample types: *1*) first and third trimester of pregnancy, mother at 3 mo postpartum, cord blood, child at 24 mo of age (*n *= 58); *2*) first and third trimester of pregnancy, mother at 3 mo postpartum, child at 24 mo of age (*n *= 77); *3*) first and third trimester of pregnancy, mother at 3 mo postpartum, cord blood (*n *= 235); *4*) first and third trimester, mother at 3 mo postpartum (*n *= 203). Missing, low-volume, or misattributed samples resulted in the combinations shown in Supplemental Figure 1. In total, *n *= 573 maternal–infant dyads were represented, with first trimester *n *= 569, third trimester *n *= 566, 3 mo postpartum *n *= 565, cord blood *n *= 295, and 24-mo-old *n *= 138, for a total of *n *= 2133 samples.

### Aflatoxin B_1_ albumin adduct measurement

#### Sample processing

AFB_1_-lysine concentrations were measured using modifications to the method reported by McCoy et al. (8). Due to volumes available and requirements for detection, 70 µL of plasma was used for samples from Malawi, while 170 µL of serum was used in samples from Bangladesh. PBS (pH 7.2) was added to bring the total volume of all samples to 200 µL. Quality control (QC) samples were processed alongside unknowns for each batch, and prepared using AFB_1_-lysine–negative pooled human donor serum (Innovative Research, Inc.) and AFB_1_-dosed rat serum (diluted with PBS to ∼13 pg AFB_1_-lysine/µL), as follows: QC_0_, 200 µL human serum, 0 µL diluted rat serum; QC_L_, 195 µL human serum, 5 µL diluted rat serum; QC_M_, 190 µL human serum, 10 µL diluted rat serum; QC_H_, 180 µL human serum, 20 µL diluted rat serum. All samples were combined with an isotopically labeled internal standard (100 µL at 5 pg AFB_1_-d_4_-lysine/µL) and 500 µL of a 6.5-mg/mL PBS solution of Pronase protease (537,088; EMD Millipore), and incubated with agitation for 18 h at 37°C. After enzymatic digestion, samples were centrifuged (3 min at room temperature at 14,000 × *g*) and the supernatant was processed on Oasis MAX solid-phase extraction 96-well plates (186,000,373; Waters), using a Positive Pressure-96 processor (186,006,961; Waters). Eluate (800 µL) was dried in a Speedvac (SPD120-15; ThermoFisher Scientific) at 35°C for 4 h, and wells were washed with 100 µL methanol, which was transferred to a V-well PCR plate. Samples were again dried in a Speedvac (35°C for 1 h), reconstituted in 40 µL of 25% aqueous methanol, and transferred to an autosampler plate (60,180–10217B; ThermoFisher Scientific; Zone-Free sealing film, ZAF-PE-50; Excel Scientific) for ultra-high-performance LC–tandem MS (UHPLC-MS/MS) analysis.

#### UHPLC-MS/MS analysis

Analysis was performed on a Vanquish Flex Quaternary UHPLC system coupled to a TSQ Quantis triple quadrupole mass spectrometer (ThermoFisher). Twenty microliters of sample was injected onto an Accucore Vanquish C18+ column (150 mm × 2.1 mm × 1.5 µm; ThermoFisher) preceded by a HyperSil GOLD C18 guard column (10 mm × 2.1 mm × 5 µm; ThermoFisher), which were held at 55°C. Samples were separated with an 18-min isocratic chromatography method, composed of water (mobile phase A), acetonitrile (B), and 0.6% aqueous formic acid (C). Initial conditions were 80% A/10% B/10%C for 1 min, stepped to 74% A/16% B/10% C and held for 7 min, followed by a step to 0% A/90% B/10% C for 3 min, and finally a step back to initial conditions, where the column was re-equilibrated for 7 min. Flow was diverted from the detector to waste from 10–14.5 min. The flow rate was held constant at 250 µL/min.

Mass spectrometry electrospray ionization source conditions were as follows: 3525 V in positive mode, sheath gas (nitrogen) 20 arbitrary units, auxiliary gas (nitrogen) 24 arbitrary units, sweep gas (nitrogen) 0 arbitrary units, ion transfer tube 350°C, vaporizer 350°C, and cone voltage 10 V. Data acquisition was via selected reaction monitoring using a collision gas pressure of 2 mTorr (argon) and the following transitions: 457.2 → 394.2 (AFB_1_-lysine), 461.2 → 398.2 (AFB_1_-d_4_-lysine). Cycle time was set at 0.35 s, resulting in a dwell time of 173 ms per transition. Collision energy and radio frequency transmission voltages were set at 21 V and 138 V, respectively, for both transitions. Q1 resolution was set at 0.7 full width at half-maximum (FWHM), and Q3 resolution was set at 1.2 FWHM.

#### Quantitation and assay performance

Peak areas were integrated automatically within TraceFinder 4.0 software (ThermoFisher Scientific), followed by visual inspection and manual integration where necessary. Quantitation was performed using an 8-point, serially diluted, isotope dilution calibration curve in 25% aqueous methanol (vol:vol). Purified synthetic AFB_1_-lysine (200 pg/µL) was diluted with 25% aqueous methanol to 45 pg/µL (calibrator 1), followed by serial 3-fold dilution to 0.021 pg/µL (calibrator 8). These calibrators were then each mixed with synthetic isotopically labeled AFB_1_-d_4_-lysine (5.0 pg/µL) in a 1:1 ratio, to create an 8-point isotope dilution calibration curve with a constant AFB_1_-d_4_-lysine concentration of 2.5 pg/µL and AFB_1_-lysine concentrations of 22.5–0.010 pg/µL. AFB_1_-lysine concentration was plotted against AFB_1_-lysine:AFB_1_-d_4_-lysine peak area ratios and a linear curve was fit with an intercept of 0 and 1/X weighted least-squares regression.

Samples were processed and analyzed in batches of 92 unknowns and 4 QCs [QC_0_ (matrix blank), QC_L_, QC_M_, and QC_H_], with separate 8-point calibration curves run in duplicate for each batch. Samples within a mother–child dyad were processed together and analyzed sequentially. In total, 26 batches were run for the Bangladesh study and 14 for Malawi samples, amounting to approximately 4500 total injections. Valid LC-MS data was available for 2107 of 2133 serum samples from Bangladesh (98.8%) and 1164 of 1202 plasma samples from Malawi (96.8%). QC sample performance across all batches was consistent: %CV for calculated AFB_1_-lysine concentrations in QC_L_ (0.33 pg/µL), QC_M_ (0.66 pg/µL), and QC_H_ (1.31 pg/µL) samples was 11.3%, 10.8%, and 10.3%, respectively. Longitudinal quantitative accuracy was within 20% of the calculated concentration for each QC level (**Supplemental Figure 2**). Across all batches, the mean slope for the 3 dilutions of QC samples was 0.421 ± 0.009 (12.0% CV), while the mean slope of the calibrators in 25% methanol was 0.424 ± 0.003 (4.5% CV), demonstrating that the presence of serum matrix did not alter quantitation (**Supplemental Figure 3**). Performance of the calibration curve was highly consistent across batches, with *R*^2^ values ranging from 0.997 to 0.999. The limit of detection (LOD) for the assay (CV ≤20%) with 200 µL input serum was 0.01 pg/µL. Experiments using 100, 80, 60, or 40 µL of serum input demonstrated assay linearity down to 0.067 pg/µL (the lowest concentration tested) with all input volumes, but >20% inaccuracy at 0.067 pg/µL with serum input ≤100 µL (**Supplemental Figure 4**).

### Data analysis

All statistical analyses were conducted separately by site. Demographic attributes of the participating populations are summarized as means ± SDs for continuous variables or count and % for categorical variables.

AFB_1_-lysine values are presented in units of pg/µL serum/plasma and reported as medians and IQRs, unless indicated otherwise. Nondetectable samples were imputed as LOD/2 (0.005 pg AFB_1_-lysine/µL). Medians and IQR values are inclusive of imputed nondetectable values, unless stated otherwise. AFB_1_-lysine adduct levels have historically been reported as adduct concentration normalized to total serum albumin (e.g., pg adduct/mg albumin), requiring the use of a separate assay and serum aliquot for total albumin quantification. As noted above, available sample volumes were limited and were less than the typical assay volume of 200 μL (Bangladesh, 170 μL; Malawi, 70 μL). In order to maximize sample availability for AFB_1_-lysine detection in the absence of prior exposure data (particularly in cord blood and infants), we did not quantify total albumin concentrations and thus are not presenting AFB_1_-lysine concentrations as normalized to total albumin.

Seasonality of sample collection in Bangladesh was defined as follows: winter, 16 October–15 February; dry, 16 February–15 June; rainy, 16 June–15 October. Seasons in Malawi were defined as follows: rainy, 16 November–15 April; dry, 16 April–15 November. These classifications are consistent with climate data reported by The World Bank (30, 31). To explore associations of AFB_1_-lysine with season within each study site, locally weighted scatterplot smoothing (LOWESS) curves were fit to AFB_1_-lysine data separately for each sample type (e.g., M-1TM, M-3TM, etc.) using either 5-point (Malawi) or 10-point (Bangladesh) smoothing, using date of sample collection as the independent variable. Differences in distributions of AFB_1_-lysine concentrations between participant groups or between season of collection were assessed by the nonparametric Kruskal-Wallis test, using the Dwass, Steel, Critchlow-Fligner multiple comparison procedure in PROC NPAR1WAY of SAS (SAS Institute). Tests were considered significant at α = 0.05.

Post hoc regression analyses of seasonal adduct accumulation and clearance rates were conducted using tobit analysis (32, 33) in PROC QLIM of SAS. The IDMS assay's LOD (0.01 pg/μL) was used as the lower-bound censoring limit, day as the independent variable (as an integer relative to the day of the year defined as the beginning of accumulation or clearance), log_10_-transformed AFB_1_-lysine adduct concentrations as the dependent variable, and repeated measurements were grouped by mother to account for within-subject variance. Within the Bangladesh dataset, only maternal data were included in this analysis, due to the limited sample size for 24-mo-old children and the high percentage of nondetectable values in cord blood samples. Data were pooled across all years of sample collection and aligned by date, irrespective of the year in which a sample was collected.

Estimates of daily intakes of AFB_1_ were calculated as in Equation 1 below, with the following variables and constants: W, body weight in kilograms; H, height in meters; BV, blood volume in liters as estimated in women by Equation 2 (34) or by body weight [estimated with weight-for-age *z* score (WAZ)] in prepubertal children (35); hematocrit (Hct), estimated by hemoglobin/3 (36, 37); 2% conversion rate of ingested AFB_1_ to AFB_1_-lysine adduct (38); 30-fold accumulation in AFB_1_-lysine adduct concentration resulting from chronic exposure relative to an equivalent single dose, assuming a 28-day lifetime of albumin (7).

(1) $$\begin{eqnarray*}
\frac{{\mu g\, AF{B_1}}}{{day}} &=& \left( {\frac{{pg\, AF{B_1}-lysine}}{{\mu L\, serum}}} \right) \times \left( {\frac{{0.68\,pg\, AF{B_1}}}{{pg\, AF{B_1}-lysine}}} \right)\nonumber\\ && \times\, BV \times Hct \times {0.02^{ - 1}} \times {30^{ - 1}}
\end{eqnarray*}$$

(2) $$\begin{eqnarray*}
BV = (0.3561 \times H^{ 3}) + (0.03308 \times W) + 0.1833
\end{eqnarray*}$$

To ascertain persistence of exposure over the time course from early pregnancy to 18 or 24 mo of child's age within maternal and infant dyads, correlation analysis and partial correlation analysis were performed among data at all time points per study site. Partial correlations were calculated between log_10_-transformed AFB_1_-lysine concentrations at each sample collection time (including imputed nondetectable values), adjusting for seasonal variability in exposure to ascertain whether some dyads were perpetually at higher risk of exposure even as environmental aflatoxin may have changed. For samples from Malawi, which had few nondetectable samples, Spearman's rank-order correlation is appropriate. Due to the higher percentage of nondetectable values in the Bangladesh dataset, correlation analysis of AFB_1_-lysine concentrations for samples from Bangladesh was also conducted using Kendall's tau-b rank-order correlation (39), which does not yield a *P* value with partial correlation analyses (40). Both correlation coefficients are presented for each site.

Finally, within the Malawi dataset, which uniquely contained data in 6-mo-old infants, regression analysis was conducted to ascertain the potential protective role of breastfeeding behaviors and other demographic factors on aflatoxin exposure in infancy. We report findings that followed an extensive model fitting exercise (41) to generate a parsimonious model relating breastfeeding, household food security, maternal parity, and season of sample collection to log_10_-AFB_1_-lysine concentrations at 6 mo of age, with β-coefficients expressed as geometric mean ratios with 95% CIs.

Statistical analysis and data visualization were performed in SAS version 9.4 (SAS Institute) and GraphPad Prism 8 (GraphPad Software).

## Results

### Participant demographics, anthropometrics, and characteristics

Features of each study sample are shown in **Table 1** and have been published in detail elsewhere (22–24, 26, 42). Within each study, participants were balanced across trial intervention groups. Compared with participating mothers in Malawi, mothers from Bangladesh were younger, shorter, weighed less, had lower BMI, had less education, and were nearly twice as likely to be primiparous. As a result of the differing study designs, Bangladeshi mothers were enrolled at the end of the first trimester (∼11 wk of gestation), while Malawian mothers’ baseline visit occurred in the middle of the second trimester (∼17 wk of gestation). Among infants, differences could be seen in child anthropometric measures, as mean values of length-for-age *z* score (LAZ), weight-for-length *z* score (WLZ), and WAZ in Bangladeshi children were all lower than those of Malawian children at each time of assessment, which was particularly notable for WLZ and WAZ. Patterns of breastfeeding practices were similar, although Bangladeshi mothers reported higher rates of exclusive breastfeeding at 3 mo postpartum (76% vs. 54%) and at 6 mo postpartum (14% vs. 7%). Homes in either setting were unlikely to have electricity or piped water, although in Bangladesh, water-sealed toilets were common. Food insecurity, albeit measured differently across studies, was considerably more severe in Malawi (82% of households reporting any level of food insecurity) than in Bangladesh (47%). These data reveal settings that challenge the well-being of mothers and infants in common and unique ways. However, descriptive data were not tested statistically between sites, given that variables were not consistently assessed in the same manner across the 2 trials.

**TABLE 1:** Participant characteristics^1^

	Bangladesh (*n* = 573)	Malawi (*n* = 255)
**Maternal**		
Intervention group		
*IFA*	292 (51.0%)	84 (32.9%)
* MMN*	281 (49.0%)	85 (33.3%)
*LNS*	—	86 (33.7%)
Age, y	23.2 (5.4)	25.1 (6.1)
Gestational age at first visit, wks	11.1 (4.5)	16.9 (2.3)
Height, cm	148.9 (5.1)	155.9 (5.6)
Weight, kg	42.9 (6.1)	53.2 (7.2)
BMI, kg/m^2^	19.4 (2.3)	21.5 (2.5)
Primiparous	209 (36.5%)	51 (20.0%)
Education^2^		
*0 y*	144 (25.2%)	75 (30.0%)
*1–4 y*	79 (13.8%)	72 (28.7%)
*5–9 y*	305 (53.2%)	89 (35.5%)
*≥10 y*	45 (7.8%)	15 (6.0%)
**Child^3^**		
Sex, male	311 (54.3%)	117 (45.9%)
Gestational age at delivery, wk	39.2 (2.7)	40.0 (1.5)
Anthropometry^4^		
LAZ		
*Birth or 1 mo*	–1.48 (1.08)	–0.89 (0.10)
*6 mo*	–1.37 (1.01)	–1.18 (1.12)
*18 or 24 mo*	–2.16 (0.98)	–1.60 (1.05)
WLZ		
*Birth or 1 mo*	–0.84 (1.02)	0.36 (1.10)
*6 mo*	–0.61 (0.99)	0.35 (1.06)
*18 or 24 mo*	–1.31 (0.91)	–0.13 (0.91)
WAZ		
*Birth or 1 mo*	–1.58 (0.96)	–0.30 (0.96)
*6 mo*	–1.37 (0.96)	–0.54 (1.13)
*18 or 24 mo*	–2.08 (0.91)	–0.85 (0.97)
Breastfeeding^5^		
3 mo of age		
*Exclusive*	429 (76.2%)	103 (53.9%)
*Predominant*	65 (11.6%)	28 (14.7%)
*Partial*	65 (11.4%)	60 (31.4%)
*None*	4 (0.2%)	0 (0.0%)
6 mo of age		
*Exclusive*	68 (13.9%)	14 (6.9%)
*Predominant*	23 (4.7%)	14 (6.9%)
*Partial*	398 (81.1%)	175 (86.2%)
*None*	2 (0.4%)	0 (0.0%)
**Household**		
Electricity	80 (14.0%)	20 (7.9%)
Piped water access	1 (0.2%)	32 (12.6%)
Toilet facility^6^		
*None*	127 (22.2%)	12 (4.7%)
*Pit latrine*	28 (4.9%)	207 (81.5%)
*Ventilated pit latrine*	—	33 (13.0%)
*Water sealed*	418 (72.9%)	—
*Flush*	0 (0.0%)	2 (0.8%)
Food security^7^		
*Food secure*	262 (53.4%)	45 (17.9%)
*Food insecure*	229 (46.6%)	206 (82.1%)

1 Values are mean (SD) or *n* (% of total). IFA, iron-folic acid; LAZ, length-for-age *z* score; LNS, lipid-based nutritional supplement; MMN, multiple micronutrient; WAZ, weight-for-age *z* score; WLZ, weight-for-length *z* score.

2 In Malawi, *n *= 4 missing educational attainment data.

3 Total numbers of respondents in Bangladesh are *n *= 556 for gestational age at delivery, *n *= 563 for LAZ at birth, *n *= 482 for LAZ at 6 mo, *n *= 484 for LAZ at 24 mo, *n *= 480 for WLZ at birth, *n *= 482 for WLZ at 6 mo, *n *= 483 for WLZ at 24 mo, *n *= 573 for WAZ at birth, *n *= 486 for WAZ at 6 mo, *n *= 500 for WAZ at 24 mo. In Malawi, *n *= 255 for gestational age at delivery, *n *= 238 for LAZ at birth, *n *= 233 for LAZ at 6 mo, *n *= 248 for LAZ at 18 mo, *n *= 234 for WLZ at birth, *n *= 233 for WLZ at 6 mo, *n *= 248 for WLZ at 18 mo, *n *= 239 for WAZ at birth, *n *= 235 for WAZ at 6 mo, *n *= 248 for WAZ at 18 mo.

4 Assessments at birth and 6 and 24 mo in Bangladesh and 1, 6, and 18 mo in Malawi.

5 Assessed in Bangladesh regarding feeding practices in the prior ∼3 mo; assessed in Malawi regarding feeding in past 24 h. Total of *n *= 563 respondents at 3 mo and *n *= 491 at 6 mo in Bangladesh; *n *= 191 at 3 mo and *n *= 203 at 6 mo in Malawi.

6 In Malawi, *n *= 1 missing toilet facility data.

7 Food insecure includes mild, moderate, or severe food insecurity. Assessed at 6 mo postpartum in Bangladesh, *n *= 491; assessed at maternal baseline enrollment in Malawi, *n *= 251.

### Aflatoxin exposure in Bangladeshi and Malawian mother–child dyads

**FIGURE 1:**
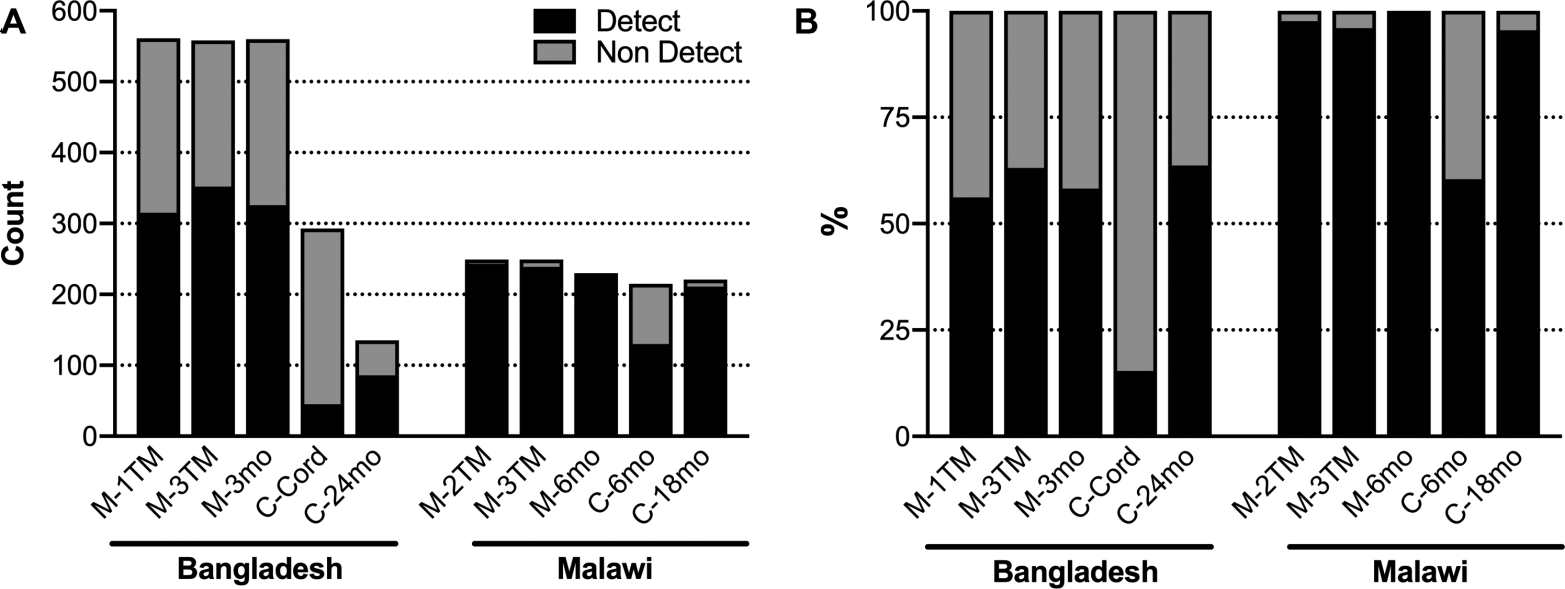
Number and percentage of samples with detectable AFB_1_-lysine adducts. (A) Counts of samples with detectable and nondetectable AFB_1_-lysine adducts. (B) Percentage of samples with detectable and nondetectable AFB_1_-lysine adducts. AFB_1_, aflatoxin B_1_; C-Cord, child's cord blood at delivery; C-6mo, child at 6 mo old; C-18mo, child at 18 mo old; C-24mo, child at 24 mo old; Detect, detectable; M-1TM, mother at first trimester; M-2TM, mother at second trimester; M-3TM, mother at third trimester; M-3mo, mother at 3 mo postpartum; M-6mo, mother at 6 mo postpartum; Non Detect, non-detectable.

Aflatoxin exposure varied substantially by study site and sample type (**Figure 1**). In Bangladesh, 53% (1124/2107) of all samples had detectable AFB_1_-lysine, while 90% (1053/1164) of samples in Malawi had detectable AFB_1_-lysine, such that it required analysis of nearly twice as many samples from the Bangladeshi women to achieve the same number of samples with detectable AFB_1_-lysine. In Bangladeshi women, the proportion of women with detectable AFB_1_-lysine was consistent across time points (59%, 993/1679), with 56% in mothers during early pregnancy (M-1TM, 315/561), 63% in mothers during late pregnancy (M-3TM, 352/558), and 58% in mothers at 3 mo postpartum (M-3mo, 326/560). Malawian mothers experienced near universal aflatoxin exposure: 98% of maternal samples overall (712/728), 98% in mothers during midpregnancy (M-2TM, 243/249), 96% in mothers during late pregnancy (M-3TM, 239/249), and 100% in mothers at 6 mo postpartum (M-6mo, 230/230). In both sites, samples collected from children 18–24 mo of age revealed similar rates of AFB_1_-lysine detection as in their mothers: in Bangladesh, 64% (86/135) of 24-mo-old children had detectable concentrations, while in Malawi, nearly all samples collected from 18-mo-old children were positive (95%, 211/221). In contrast, in Malawi, only 60% (130/215) of samples collected from 6-mo-old children were positive for AFB_1_-lysine. Cord blood samples collected from Bangladeshi pregnancies rarely contained detectable levels of AFB_1_-lysine (15%, 45/293).

**FIGURE 2:**
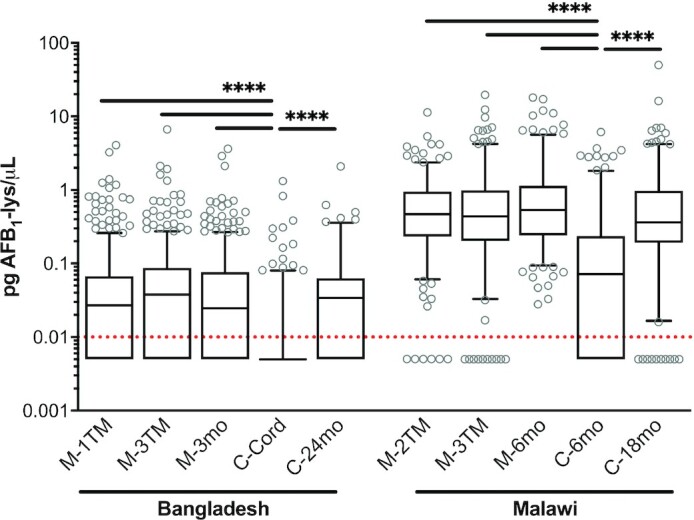
Distributions of AFB_1_-lysine (lys) adduct levels within Bangladeshi and Malawian mother–child dyads. Box plots of AFB_1_-lysine adduct concentration with whiskers extended to 5th and 95th percentiles. Dotted red line indicates limit of detection (0.01 pg/µL); nondetectable values are imputed at 0.005 pg/µL. Horizontal bars indicate statistical significance in pairwise comparisons: ^****^P < 0.0001 by Kruskal-Wallis test and Dwass, Steel, Critchlow-Fligner adjustment for multiple comparisons. AFB_1_, aflatoxin B_1_; C-Cord, child's cord blood at delivery; C-6mo, child at 6 mo old; C-18mo, child at 18 mo old; C-24mo, child at 24 mo old; M-1TM, mother at first trimester; M-2TM, mother at second trimester; M-3TM, mother at third trimester; M-3mo, mother at 3 mo postpartum; M-6mo, mother at 6 mo postpartum.

Concentrations of AFB_1_-lysine were distributed log-normally and followed similar trends as those observed in AFB_1_-lysine detection prevalence: higher AFB_1_-lysine concentrations in Malawi than in Bangladesh, approximately equivalent concentrations in mothers and children at 18 or 24 mo, but lower concentrations in the perinatal period (cord blood, Bangladesh) or infancy (6 mo, Malawi) than later in childhood (**Figure 2**). Overall, mothers in Malawi had median AFB_1_-lysine concentrations of 0.469 pg/µL (IQR: 0.225–1.027), while median concentrations in Bangladeshi mothers were nearly 16-fold lower, at 0.030 pg/µL [not detected (n.d.)–0.077]. Among Malawian mothers, AFB_1_-lysine concentrations were consistently high in the second trimester (0.466 pg/µL, 0.232–0.937), third trimester (0.439 pg/µL, 0.206–0.981), and 6 mo postpartum (0.536 pg/µL, 0.241–1.135). Although substantially lower in magnitude than in Malawi, distributions in Bangladeshi mothers were also similar in the first trimester (0.027 pg/µL, n.d.–0.066), third trimester (0.038 pg/µL, n.d.–0.087), and at 3 mo postpartum (0.025 pg/µL, n.d.–0.076). By 18 and 24 mo of age, AFB_1_-lysine concentrations in children from Bangladesh (24 mo of age; 0.034 pg/µL, n.d.–0.063) and Malawi (18 mo; 0.370 pg/µL, 0.195–0.964) did not differ from maternal values. However, AFB_1_-lysine concentrations were lower in cord blood of Bangladeshi infants and 6-mo-olds from Malawi (0.072 pg/µL, n.d.–0.236) than at any other time point in each respective population (*P *< 0.0001 for all comparisons). In Bangladesh, AFB_1_-lysine was undetectable in 85% of cord blood samples, and among the 15% of cord blood samples that had detectable AFB_1_-lysine concentrations, the median (IQR) concentration was 0.045 pg/µL (0.031–0.088).

**FIGURE 3:**
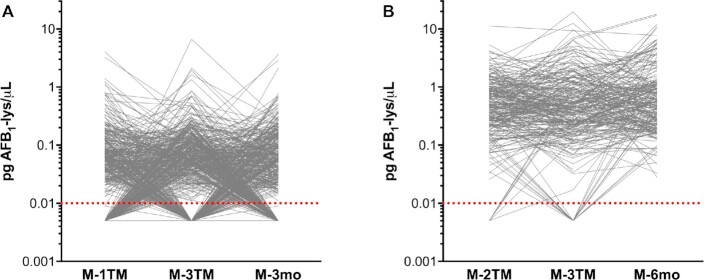
Subject-specific trajectories of AFB_1_-lysine (lys) adduct concentrations within Bangladeshi and Malawian women through pregnancy and into the postpartum period. Data as spaghetti plots of AFB_1_-lysine adduct concentration in Bangladeshi (A) and Malawian (B) women. Gray lines connect samples from a single individual. Dotted red lines indicate limit of detection (0.01 pg/µL); nondetectable values are imputed at 0.005 pg/µL. AFB_1_, aflatoxin B_1_; M-1TM, mother at first trimester; M-2TM, mother at second trimester; M-3TM, mother at third trimester; M-3mo, mother at 3 mo postpartum; M-6mo, mother at 6 mo postpartum.

Examination of mother-specific AFB_1_-lysine trajectories revealed distinct patterns of exposure in Bangladesh and Malawi (**Figure 3**). Mothers in Bangladesh experienced substantial within-subject variability in AFB_1_ internal dose across time (Figure 3A), as longitudinal sampling revealed groups of subjects alternating between nondetectable and detectable values (0.065, 0.038–0.130 pg/µL). In contrast, within Malawian mothers, there was a greater persistence of AFB_1_-lysine at a higher magnitude over time (Figure 3B).

### Association of season with aflatoxin exposure

**FIGURE 4:**
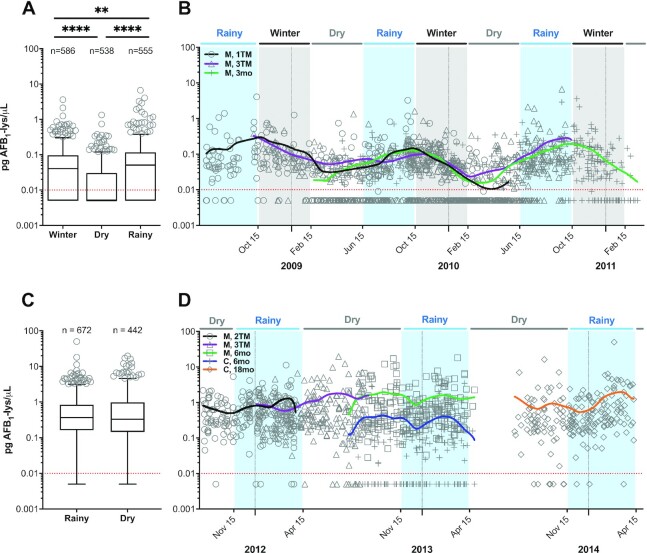
Seasonality of AFB_1_-lysine (lys) adduct exposure in Bangladesh and Malawi. (A) Boxplot of AFB_1_-lysine adduct concentrations in Bangladeshi mothers, for samples collected during the winter, dry, or rainy seasons. Whiskers extend to the 5th and 95th percentiles. Dotted red line indicates limit of detection (0.01 pg/µL); nondetectable values are imputed at 0.005 pg/µL. (B) AFB_1_-lysine adduct concentrations in Bangladeshi mothers, plotted by date of sample collection. LOWESS curves were fit to data from each maternal sample type. (C) Boxplot of AFB_1_-lysine adduct concentrations in Malawian mothers and children, for samples collected during either the rainy or dry seasons. Data are plotted as in panel A. (D) AFB_1_-lysine adduct concentrations in Malawian mothers and children, plotted by date of sample collection. LOWESS curves were fit to data from each sample type. Horizontal bars in boxplots indicate statistical significance pairwise comparisons by Kruskal-Wallis test, using the Dwass, Steel, Critchlow-Fligner adjustment for multiple comparisons; **P < 0.01, ^****^P < 0.0001. AFB_1_, aflatoxin B_1_; C, 6mo, child at 6 mo old; C, 18mo, child at 18 mo old; LOWESS, locally weighted scatterplot smoothing; M, 1TM, mother at first trimester; M, 2TM, mother at second trimester; M, 3TM, mother at third trimester; M, 3mo, mother at 3 mo postpartum; M, 6mo, mother at 6 mo postpartum.

Based on AFB_1_-lysine trajectories in Bangladeshi mothers (Figure 3A), we suspected that seasonal differences in aflatoxin exposure could explain the observed within-subject variance. As shown in **Figure 4**, we examined the seasonality of AFB_1_-lysine concentrations in both Bangladesh and Malawi. Regardless of maternal pregnancy status, the Bangladeshi dry season was associated with significantly lower serum AFB_1_-lysine concentrations (n.d.; IQR: n.d.–0.030; 37% detectable) than either the winter (0.041, n.d.–0.097; *P* < 0.0001; 65% detectable) or rainy seasons (0.051, n.d.–0.117; *P* < 0.0001; 74% detectable) (Figure 4A). Figure 4B depicts a LOWESS analysis of serum AFB_1_-lysine concentration plotted against date of sample collection (spanning ∼2.8 y from June 2008 to March 2011) and demonstrates a seasonal oscillation in AFB_1_-lysine concentrations, with an annual nadir shortly after the end of winter and an apex in mid-October, at the end of the rainy season.

In contrast to Bangladesh, we detected no significant differences between distributions of AFB_1_-lysine concentrations in maternal and child samples collected during Malawi's rainy (0.368, 0.162–0.844; 98% detectable) compared with dry (0.330, 0.145–0.986; 91% detectable) seasons (Figure 4C). This was consistent with LOWESS analysis, which revealed no seasonal patterns in AFB_1_ exposure among Malawian mother–child dyads across a period of approximately 2.7 y (September 2011–April 2014; Figure 4D) and suggests that there is consistent exposure to aflatoxin throughout the year in Malawi.

**FIGURE 5:**
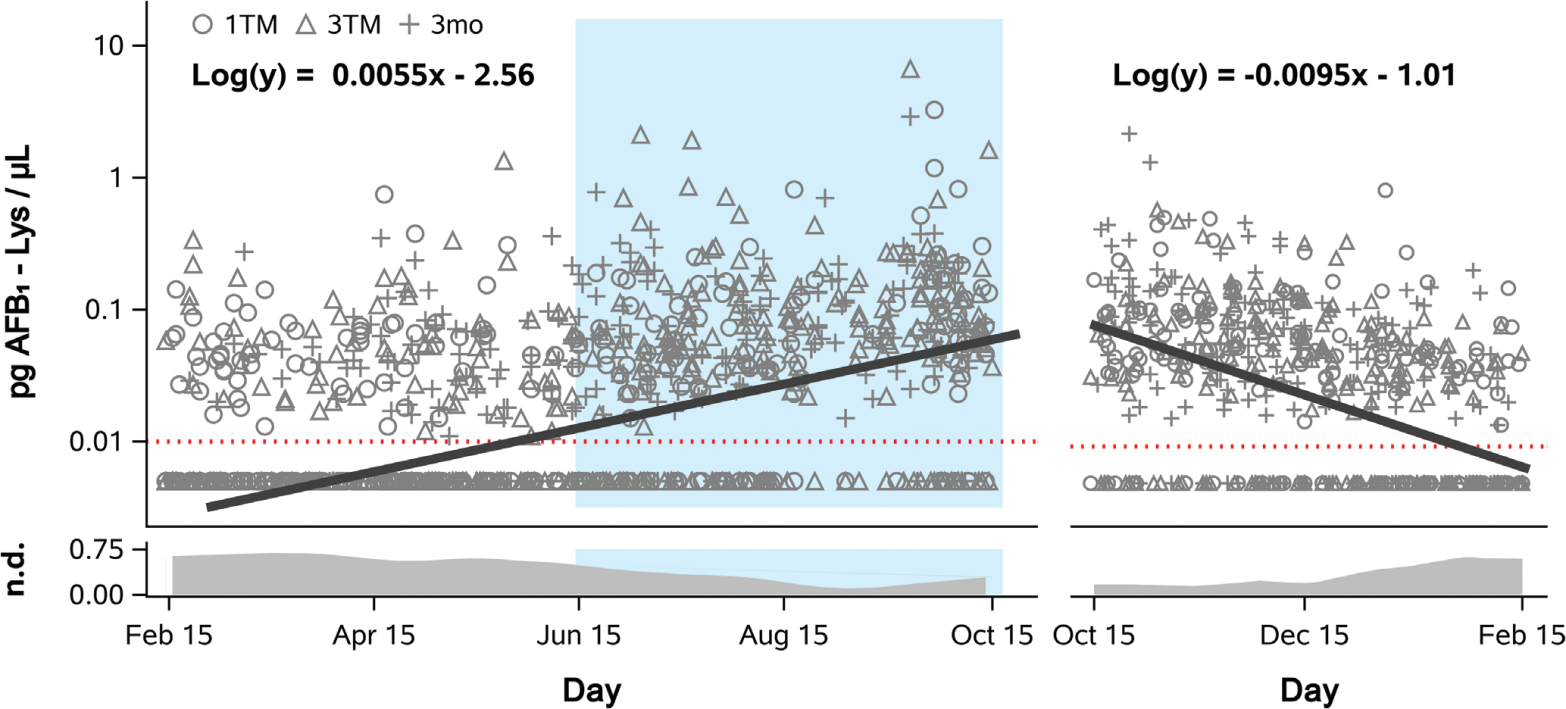
Rates of seasonal change in AFB_1_-lysine (Lys) internal dose in Bangladeshi women. Censored linear regression of AFB_1_-lysine adduct concentration (log_10_-transformed) compared with day of sample collection during the dry and rainy seasons (left) or winter season (right), across all maternal sample types (1TM, 3TM, 3mo) and years of sample collection (2008–2011). Blue shading indicates the timing of the rainy reason. Units of the independent variable was the number of elapsed days relative to 15 February in the dry and rainy seasons (242 days total) or relative to 15 October in the winter season (123 days total). Censored regression best-fit lines and equations are shown for dry + rainy and winter seasons separately. Both slopes are significantly different from 0 (*P* < 0.0001). Dotted red line indicates limit of detection (0.01 pg/µL); nondetectable values were imputed at 0.005 pg/µL. The daily proportion of samples with nondetectable AFB_1_-lysine concentrations is shown in the band plots at the bottom of the graph; data represent LOWESS curves fit to daily values. AFB_1_, aflatoxin B_1_; LOWESS, locally weighted scatterplot smoothing; n.d., not detected; 1TM, first trimester; 3mo, 3 mo postpartum; 3TM, third trimester.

We next sought to quantify the rates of AFB_1_-lysine adduct accumulation and clearance in Bangladeshi mothers. Per our LOWESS analyses (Figure 4A, B), we defined the period of accumulation to encompass the Bangladeshi dry (15 February to 15 June) and rainy (15 June to 15 October) seasons (242 days total), while adduct clearance occurred during the winter (15 October to 15 February; 123 days). Using censored regression analysis on log_10_-transformed AFB_1_-lysine values, which facilitated the estimation of adduct concentrations below the assay's LOD (0.01 pg/μL), geometric mean AFB_1_-lysine adduct concentrations in Bangladeshi mothers accumulated from an estimated nadir of 0.003 pg/µL on 15 February to a peak of 0.060 pg/µL on 15 October (**Figure 5**, left). Similarly, examining the clearance of adducts across 3 winter seasons revealed a peak of 0.099 pg/µL on 15 October and an estimated nadir of 0.007 pg/µL on 15 February (Figure 5, right). Averaged across both the accumulation and clearance periods, arithmetic mean peak (15 October) and nadir (15 February) concentrations were thus 0.080 and 0.005 pg/μL, respectively. Using these average values, the rates of accumulation and clearance were estimated as 0.310 and 0.610 fg/μL/day, respectively. Note that, based on albumin adduct clearance kinetics (43), only ∼1% of AFB_1_-adducted albumin resulting from exposures at the end of the rainy season (peak exposure) would be estimated to remain in circulation 123 days later (winter nadir).

Translating our measurements of AFB_1_ internal dose (reflected by the AFB_1_-lysine biomarker) to group-level estimated dietary intake of AFB_1_ via Equation 1, plasma AFB_1_-lysine concentrations in Malawian mothers (0.469 pg/μL) reflect a daily average dietary intake of approximately 648 ng AFB_1_/day (12.2 ng/kg/day). This is contrasted with exposure in Bangladesh, where year-round median AFB_1_-lysine concentrations (0.030 pg/μL) result in estimated intakes of 37 ng AFB_1_/d and 0.86 ng/kg/day.

Accounting for the seasonality of exposure observed in Bangladesh, estimated AFB_1_-lysine concentrations peaked at the end of the rainy season (0.080 pg/µL, per censored regression) and were at their lowest at the end of winter (0.005 pg/µL), translating to an estimated daily total intake of 98 and 6.14 ng AFB_1_/day (2.3 and 0.14 ng/kg/day), respectively.

Estimated AFB_1_ exposure in children (Bangladesh, 24 mo of age, 0.034 pg/μL; Malawi, 18 mo of age, 0.370 pg/μL) revealed a lower daily average total intake than in their mothers: 14 ng/day in Bangladeshi children (without accounting for seasonality) and 141 ng/day in Malawi. However, after normalizing to body weight, estimated child exposures were similar to maternal exposures at each site: 1.5 ng/kg/day in 24-mo-old Bangladeshi children and 14.1 ng/kg/day in Malawian children at 18 mo of age.

### Persistence and determinants of exposure

Bivariate correlation analysis of AFB_1_-lysine concentrations between times of sample collection within women and mother–infant dyads, without adjustment for seasonality, is shown in **Supplemental Figure 5**. Results were not substantially altered after adjustment for season of sample collection with partial correlation analysis, as seen in **Table 2**. Accounting for the variability contributed by season did not substantially alter the strength of associations in aflatoxin exposure observed between time points in Bangladesh or, as expected given the lack of seasonality in exposure, in Malawi. In Bangladesh, associations were weaker than those observed in Malawi, but that they were positive (as assessed by Kendall's tau-b rank-order correlation test) in all but 1 case (M-3TM and M-3mo) suggests that certain dyads were at a persistently greater risk of exposure even as seasonal influences on exposure varied. Cord blood AFB_1_-lysine, despite being low in detection prevalence and concentration, was associated with maternal concentrations at the times surrounding its collection (τ = 0.169 for M-3TM and τ = 0.228 at M-3mo), while values in children at 24 mo showed modest but positive associations with prior values despite the extensive time interval between periods of sample collection. In mothers from Malawi, the strongest correlations were observed between samples collected during midpregnancy (M-2TM) and those collected either during the third trimester (M-3TM; *ρ* = 0.40, *P* < 0.0001) or 6 mo postpartum (M-6mo;  *ρ* = 0.30, *P* < 0.0001). Malawian maternal concentrations during the third trimester were less strongly correlated with concentrations measured 6 mo after delivery (*ρ* = 0.26, *P* < 0.005). Additionally, a strong correlation was observed between maternal and child AFB_1_-lysine concentrations measured in samples collected at the same study visit, 6 mo after delivery (*ρ* = 0.63, *P* < 0.0001). Concentrations in samples collected from children at 18 mo of age were less strongly correlated with concentrations at 6 mo of age (*ρ* = 0.25, *P* < 0.005) and were not correlated with concentrations measured in their mothers at any point during or after pregnancy.

**TABLE 2:** Nonparametric partial correlation matrix for association of aflatoxin exposure between sample types among maternal–infant dyads from pregnancy through 24 or 18 mo of age in Bangladesh and Malawi^1^

Bangladesh					Malawi				
	**M-1TM**	**M-3TM**	**M-3mo**	**C-Cord**		**M-2TM**	**M-3TM**	**M-6mo**	**C-6mo**
**M-3TM**	*r_s_* = 0.110				**M-3TM**	*r_s_* = 0.375			
	*P = *0.010					*P < *0.0001			
	*τ* = 0.075					*τ* = 0.263			
	*n* = 549					*n* = 219			
**M-3mo**	*r_s_* = 0.131	*r_s_* = –0.004			**M-6mo**	*r_s_ *= 0.293	*r_s_* = 0.263		
	*P = *0.002	*P* = 0.918				*P < *0.0001	*P < *0.0001		
	*τ* = 0.100	*τ* = -0.006				*τ* = 0.202	*τ* = 0.181		
	*n* = 549	*n* = 547				*n* = 224	*n* = 224		
**C-Cord**	*r_s_* = 0.103	*r_s_* = 0.197	*r_s_* = 0.270		**C-6mo**	*r_s_* = 0.161	*r_s_* = 0.139	*r_s_* = 0.626	
	*P* = 0.086	*P = *0.001	*P < *0.0001			*P = *0.021	*P = *0.046	*P < *0.0001	
	*τ* = 0.090	*τ* = 0.169	*τ* = 0.228			*τ* = 0.111	*τ* = 0.097	*τ* = 0.476	
	*n* = 283	*n* = 282	*n* = 281			*n* = 208	*n* = 208	*n* = 214	
**C-24mo**	*r_s_* = 0.122	*r_s_* = 0.192	*r_s_* = 0.215	*r_s_* = 0.264	**C-18mo**	*r_s_* = 0.054	*r_s_* = 0.079	*r_s_* = 0.172	*r_s_* = 0.246
	*P* = 0.169	*P = *0.027	*P = *0.016	*P = *0.050		*P* = 0.429	*P* = 0.249	*P = *0.011	*P = *0.0004
	*τ* = 0.104	*τ* = 0.134	*τ* = 0.154	*τ* = 0.216		*τ* = 0.037	*τ* = 0.055	*τ* = 0.115	*τ* = 0.174
	*n* = 130	*n* = 135	*n* = 127	*n* = 58		*n* = 215	*n* = 216	*n* = 220	*n* = 205

1 Data shown are the Spearman rho (*r_s_*) or Kendall's tau-b (*τ*) partial correlation coefficients (adjusting for season of sample collection), *P* value for the Spearman partial correlation (*P*), and the number of available samples for each comparison (*n*). As the partial Kendall distribution is unknown, *P* values were not available for Kendall partial correlation analysis (40). C-Cord, child's cord blood at delivery; C-6mo, child at 6 mo old; C-18mo, child at 18 mo old; C-24mo, child at 24 mo old; M-1TM, mother at first trimester; M-2TM, mother at second trimester; M-3TM, mother at third trimester; M-3mo, mother at 3 mo postpartum; M-6mo, mother at 6 mo postpartum.

Given the dramatically lower AFB_1_ exposure in 6-mo-old children in Malawi than at any other sample collection time point, we sought to determine via multivariate regression analysis whether infant feeding mode could be a protective factor. In line with a hypothesized protective nature of exclusive breastfeeding, we demonstrated a 58% reduction (*P* = 0.010) in AFB_1_-lysine concentrations among 6-mo-old infants who were exclusively breastfed at 3 mo of age compared with other modes of feeding (**Table 3**). Additionally, children from households experiencing any degree of food insecurity (mild, moderate, or severe) exhibited 2.18-fold greater AFB_1_-lysine concentrations (95% CI: 1.16-, 4.10-fold) than children from food-secure households.

**TABLE 3:** Multiple regression of factors influencing AFB_1_ exposure in 6-mo-old Malawian children^1^

	Geometric mean ratio of AFB_1_-lysine concentration (95% CI)	*P*
Exclusively breastfed at 3 mo of age^2^	0.42 (0.22, 0.82)	0.010
Food insecure^3^	2.18 (1.16, 4.10)	0.016
Mother primiparous^4^	0.52 (0.26, 1.03)	0.059
Sample collected during dry season^5^	0.56 (0.31, 1.01)	0.056

1 AFB_1_, aflatoxin B_1_.

2 Compared with predominant, partial, or no breastfeeding at 3 months of age.

3 Mild, moderate, or severe household food insecurity vs. food secure.

4 Compared with multiparous.

5 Compared with rainy season.

## Discussion

In this report, we present data from one of the largest longitudinal analyses of aflatoxin exposure throughout pregnancy and early childhood, representing over 800 mother–child dyads across 2 nutrition intervention trials, in settings where nutritional risks to mothers and infants are common, yet dietary patterns and food insecurity vary. We found striking and persistent levels of maternal aflatoxin exposure in rural Malawi, with nearly 100% prevalence and substantial plasma AFB_1_-lysine concentrations, while exposure in Bangladeshi mothers was less prevalent, at approximately 60% of all maternal samples, and was up to 1 order of magnitude lower in concentration. However, these summary values belied significant seasonal variability in Bangladesh, with a nearly 3-fold annual dynamic range in AFB_1_-lysine concentrations and large shifts in detection prevalence across the year. Finally, while offspring seemed to be relatively protected from aflatoxin exposure *in utero* and through breastfeeding early in infancy (at 6 mo of age), children aged 18 mo (Malawi) or 24 mo (Bangladesh) experienced rates and magnitudes of aflatoxin exposure that were comparable to those in their mothers.

Before comparing findings with those of previous studies, it is important to note that we report AFB_1_-lysine concentrations in volumetric terms (e.g., pg/µL), rather than relative to total serum albumin concentrations (e.g., pg/mg albumin). Historically, expressing AFB_1_-lysine values relative to serum albumin—which can be determined through various colorimetric assays or immunoassays in a serum aliquot separate from that used for AFB_1_-lysine quantification—has been the conventional practice. However, while that approach was warranted to normalize AFB_1_-lysine values in older methods (44, 45), this is not necessary with IDMS (8), which is now the gold-standard method for measuring AFB_1_-albumin adducts and was the approach utilized in this report. Due to the use of isotopically labeled AFB_1_-lysine internal standards and tandem MS quantification, the IDMS approach can directly quantify serum or plasma concentrations of AFB_1_-lysine adducts, providing an accurate metric of AFB_1_ internal dose (46). Furthermore, due to the variance and bias across albumin assay formats and manufacturers (47), presenting AFB_1_-lysine values as normalized to serum albumin, rather than volumetrically, complicates harmonization and conversion of internal dose to exposure estimates, as in Equation 1 above. Other groups have also questioned the utility of albumin normalization for AFB_1_-lysine measurements (48). However, for the purposes of comparison to previously published, albumin-normalized results, a rough conversion to units of pg/mg albumin can be achieved through multiplication of values expressed in pg/µL by a factor of 23.8 (based on a population average albumin concentration of 42 mg/mL). We will discuss our data in the context of prior work by presenting estimated albumin-normalized values alongside our volume-normalized results, as well as with appropriate conversions to account for known quantitative biases of other AFB_1_-lysine analytical methods (49). Using these conversions, the level of exposure we observed in Bangladesh is very similar to those reported recently from Nepal by Andrews-Trevino et al. (18), with medians of 1.20 pg/mg in maternal samples during pregnancy (equivalent to ∼0.053 pg/μL), 0.72 pg/mg in 3-mo-old children (0.032 pg/μL), and 1.11 pg/mg at 18–22 mo (0.049 pg/μL). Similarly, we report levels of exposure in Malawi mothers, which, after conversion, are similar to maternal concentrations in other African populations, such as Ghana (mean, 10.9 pg/mg; equivalent to 0.481 pg/μL) (50) and Uganda (median, 5.83 pg/mg; equivalent to 0.256 pg/μL) (51), while being higher than concentrations recently reported in pregnant Rwandan women (median, 1.7 pg/mg; equivalent to 0.071 pg/μL) (52).

In Latin America, where maize consumption is engrained within the culture and provides nearly 20% of energy intake, we have shown that tortillas account for over half of the total maize intake and their consumption is dose-dependently associated with elevated AFB_1_-lysine concentrations (53). Additionally, in Qidong, China, our group has shown that, driven by economic factors, a widespread dietary transition from maize to rice, which is less commonly contaminated, resulted in a dramatic drop in AFB_1_-lysine concentrations and a precipitous decline in hepatocellular carcinoma incidence (54). In Malawi, maize comprises nearly two-thirds of energy intake among pregnant women (55), resulting in a diet with a very low degree of diversity and high contribution from one of the staple crops most commonly contaminated by aflatoxins (46). With complementary foods that mimic the adult diet, complementary feeding in Malawi places children at high risk of aflatoxin exposure, such that it is not surprising that children at 18 mo of age experienced approximataely the same level of aflatoxin exposure as their mothers. In a bivariate analysis among 6-mo-old infants in the current study, consumption of *nsima*, a thick, maize-based porridge, was associated with 47% (95% CI: 1%, 114%) increased risk of AFB_1_ exposure, while consumption of *likuni*, a commercially produced, liquid maize-soy blended porridge, reduced exposure by 32% (95% CI: 8%, 50%). While these associations were no longer statistically significant after adjustment for other covariates (41), they suggest that replacing poor-quality staples with higher-quality alternatives might be necessary to reduce the burden of aflatoxin exposure during pregnancy and complementary feeding in settings like Malawi, where exposure is persistent. This approach is supported by results from interventions such as the one by Hoffman et al. (56) in a community in eastern Kenya, in which the investigators facilitated the replacement of contaminated maize with clean maize, resulting in a reduced risk of stunting among children at ∼1 y of age. This represents one strategy to reduce aflatoxin exposure in settings where monotonous diets rely on persistently contaminated foodstuffs.

We observed a statistically significant 2.18-fold increase in circulating AFB_1_-lysine adduct concentrations among 6-mo-old Malawian children in food-insecure households compared with counterparts in food-secure households. This is, to our knowledge, the first report of such an association between food insecurity and aflatoxin exposure. Moreover, given the very high rates of household food insecurity in this population (82%), this association is particularly troubling and suggests that food insecurity may play a significant role in driving aflatoxin exposure in Malawi. Previous research from several authors of this paper has shown an inverse association between food insecurity and dietary diversity in pregnant and lactating women in Malawi (57). Poor dietary diversity has been shown to be related to the magnitude of aflatoxin exposure and increasing dietary diversity is a valuable strategy for reducing exposure to aflatoxins (4). Thus, whether strategies focus on increasing dietary diversity or on reducing household food insecurity—such as by provision of LNSs (58)—a diminished dietary reliance on maize is likely to reduce aflatoxin exposure in Malawi.

In contrast to dietary patterns in Malawi, rice is the staple grain in Bangladesh. Additionally, food insecurity was lower in our sample of Bangladeshi women (47%) than in Malawi (82%), while dietary diversity was higher: foods such as fish were consumed frequently (∼65% reported consuming at least 3 times weekly), while other meat, dairy, eggs, and yellow or green vegetables were consumed by at least 15% of respondents at least 3 times weekly (24). Work from Nguyen et al. (59) supports the notion that Bangladeshi women have a relatively diverse diet, in that the majority of pregnant women reported consuming foods from 5 different food groups within the past 24 h. Thus, while a monotonous maize-based diet likely explains the high, persistent exposure patterns observed in Malawi, greater dietary diversity in Bangladesh may explain the comparatively low overall exposure in Bangladesh. However, previous work from Stevens et al. (60) has shown that dietary diversity among pregnant women varies by season in Bangladesh, being lowest in the dry spring and summer, but highest during the late autumn and winter. In line with this, we found that aflatoxin exposure increases from 15 February through 15 October, particularly during the rainy season (15 June to 15 October), while declining during the winter, from 15 October to 15 February. A previous report from Bangladesh has shown a similar seasonal pattern of aflatoxin exposure, with the highest exposures at the end of the rainy season (61). Thus, aflatoxin exposure in Bangladesh may closely trail seasonal fluctuations in dietary diversity, such that aflatoxin exposure rises following periods of low dietary diversity and begins falling after dietary diversity reaches its yearly peak. In the present study, the rate of adduct clearance in Bangladeshi mothers during the winter season was 2-fold greater than the rate of accumulation during the dry and rainy seasons, while estimated maternal daily aflatoxin intake at the end of the winter was 94% lower than estimated peak intake after the rainy season. These data reveal a precipitous annual reduction in aflatoxin exposure in this population. The report on pregnant Bangladeshi women by Stevens et al. (60) also revealed significant increases in the consumption of meat, poultry, and fish beginning in late autumn and continuing through the winter—aligning with the rapid decline we observe in aflatoxin internal dose. However, it is unclear whether these foods are directly displacing aflatoxin-contaminated foods from the diet or are surrogate indicators of more broad dietary changes. Future analyses will need to assess whether seasonal consumption of specific foods may enhance or mitigate aflatoxin exposure in this sample of Bangladeshi mothers. As in Malawi, interventions that encourage dietary diversity may present a feasible and economical strategy for primary exposure prevention in Bangladesh.

Patterns of exposure in Bangladesh and Malawi were used to ascertain estimates of aflatoxin intakes. In Malawian women, estimates of intake were ∼650 ng/day year-round, with estimates in Bangladeshi women ranging from 98 ng/day in the season of highest exposure to 6 ng/day when exposure was lowest. Intakes in Malawi are consistent with a persistent, single source of aflatoxin in a food-insecure environment, but identifying and mitigating transient sources of exposure in Bangladesh could be more challenging. Calculations also demonstrate that, despite a lower total intake of aflatoxin in children than adults (∼3- to 5-fold lower), after normalizing to body weight, estimated intakes in both Bangladeshi and Malawian children [1.5 ng/kg/day (annual) and 14.1 ng/kg/day, respectively] were slightly higher than those of women in the same setting [0.86 ng/kg/day (annual) and 12.2 ng/kg/day, respectively]. It should be noted that these calculations represent group averages, while more refined estimates at the individual level could also be done to reveal the variance of intake in these environments.

As reported elsewhere (62), while AFB_1_ is known to cross the human placenta, the fetus appears to be somewhat protected, with approximately 25% of the maternal dose reaching the fetal circulation. In Bangladesh, although aflatoxin exposure varied by season, maternal exposures during late pregnancy and at 3 mo postpartum were modestly associated with cord blood aflatoxin adduct concentrations. Caution is warranted in interpreting the maternal–cord blood association due to the high rate of AFB_1_-lysine assay detection-limit censoring in cord blood samples (39), but comparing AFB_1_-lysine detection prevalence in maternal samples (60%, late pregnancy and 3 mo postpartum combined) and cord blood (15%) does support the hypothesis that the fetus is protected from a proportion of the maternal AFB_1_ exposure. Additionally, we found that while the median circulating AFB_1_-lysine concentrations in mothers at 32 wk of gestation was 0.038 pg/µL, the median concentration in cord blood samples was at least ∼4-fold lower, below our assay's LOD of 0.010 pg/µL. These results are in line with data from western Africa, which showed a modest correlation (Spearman's *ρ* = 0.383) between maternal AFB_1_-lysine concentrations during pregnancy (100% detectable) and those in cord blood samples (49% detectable), as well as cord blood concentrations that were approximately 25% of those found in the maternal circulation during pregnancy (10.1 and 40.4 pg/mg, respectively; equivalent to ∼0.085 and 0.34 pg/μL, respectively) (63). While cord blood samples were not collected from Malawian pregnancies in the present study, exposure in Malawian mothers at 36 wk of gestation (M-3TM; 96% detectable, 0.439 pg/μL) was comparable to values reported in western Africa, suggesting that fetal exposure in Malawi may follow similar trends. Assuming that only 25% of the maternal AFB_1_ dose is transferred to the fetal circulation, it is likely that significant *in utero* AFB_1_exposure would still be highly prevalent in the Malawi population.

While fetal protection from maternal aflatoxin exposure while *in utero* is governed by placental physiology, protection of infants from exposure after birth is, in large part, dependent upon breastfeeding and complementary feeding practices. Breastfeeding appeared to be protective against AFB_1_ exposure in Benin and Togo (64, 65) and Bangladesh (61), although not in Nepal (66). We found that exclusive breastfeeding at 3 mo postpartum was associated with a 58% reduction in AFB_1_-lysine concentrations in 6-mo-old Malawian infants, relative to other modes of feeding. Notably, rates of exclusive or predominant breastfeeding among Malawian mothers were relatively high at 3 mo postpartum (53.9% exclusive, 68.6% exclusive or predominant) but very low by 6 mo postpartum (6.9% and 13.8%, respectively), suggesting that most children received protection from exposure during only a short window early in life. Coincidentally, these data highlight a significant benefit of the AFB_1_-lysine biomarker for assessing exposure prevention or mitigation interventions—the long half-life of the albumin adduct allows detection of the effects of a protective behavior 3 mo after its occurrence, despite rates of the protective behavior having fallen to ineffective levels at the time of biomarker measurement. Use of biomarkers with shorter residence times—urinary metabolites (67, 68), for example—would require much more frequent sampling to dissect the early compared with late trends.

It is important to note that aflatoxins can be transferred from mother to child through breast milk. Aflatoxin M_1_ (AFM_1_) is a toxic and carcinogenic hydroxylated metabolite of AFB_1_ found in breast milk of aflatoxin-exposed women (46, 69), which has been found to be inversely associated with HAZ and WAZ in several studies (16). Moreover, AFB_1_ itself can be found in breast milk, sometimes at concentrations higher than AFM_1_ (70). While promoting recommended breastfeeding practices can be a valuable strategy to reduce AFB_1_ exposure in infants (as well as for achieving the established nutritional and immunological benefits of breast milk), it may not eliminate aflatoxin exposure even in exclusively breastfed infants, and it does not mitigate aflatoxin exposure and the attendant health risks within mothers. We did not assess breast milk for aflatoxin content and therefore cannot ascertain whether infants in our study were exposed to AFM_1_ through breastfeeding or the proportion of infant AFB_1_ exposure that may occur through the breast milk.

This study was motivated by the growing body of evidence suggesting that aflatoxin exposure *in utero* or during critical windows early in life may lead to harmful and potentially irreversible impacts on child development (2, 4). Aflatoxin exposure and its effects on child growth have been reported extensively in west African populations, with most investigations in this region demonstrating that either maternal or child exposures are associated with reduced birth weight (50), WAZ (63, 71), LAZ/HAZ (63, 65, 71), and/or WLZ (71). In contrast, studies exploring these questions in eastern Africa and southeast Asia have produced conflicting results both supporting (18, 19, 51) and not supporting (66, 72–75) a role for aflatoxin in fetal growth restriction and childhood stunting. However, those studies that do not control for key determinants of aflatoxin exposure—such as seasonality and breastfeeding, as we show in the present report—may bias results towards the null (61, 75), while other studies with similar sample sizes, populations, and exposures may reveal an effect of aflatoxin on growth parameters due to more complete statistical accounting of variance components (71). In fact, most of the studies that have failed to find an association between aflatoxin exposure and child growth outcomes have had small sample sizes, have not adjusted for key variables such as seasonality or breastfeeding practices, or both (66, 72–75). Moreover, while some have suggested that null results may have been the result of low aflatoxin exposures, perhaps below a critical threshold for biological effect (75), the recently published study in Nepalese subjects from Andrews-Trevino and colleagues (18) revealed highly significant reductions in postnatal LAZ, WAZ, WLZ, length, and knee-heel length in association with aflatoxin exposures that were lower than those in many of the prior studies that have reported no association. Notably, the AFB_1_-lysine concentrations that Andrews-Trevino et al. reported in Nepal are similar to those we measured in Bangladeshi mothers and children. The statistically significant results reported by Andrews-Trevino et al. are likely not only due to a larger sample size (*n *= 1675) than most prior investigations but also through controlling for seasonal timing of sample collection, which we and others have shown to be a major factor influencing aflatoxin intake in southeast Asia. Of note, in the present study, growth characteristics of infants were poorer in Bangladesh than in Malawi, despite higher aflatoxin exposure in the latter. Thus, aflatoxin exposure is but one potential etiological contributor to poor growth, with a role that is likely context-specific and challenging to disentangle from contributions of poor diet overall. Nonetheless, the findings here provide a foundation for further explorations of aflatoxin exposure on maternal and child outcomes, such as growth, which can be compared across settings.

This study has many strengths. The sample size, over 800 mother–child dyads across both sites, is among the largest longitudinal aflatoxin biomonitoring studies of its kind. Moreover, the diversity of maternal and child characteristics, magnitude and prevalence of exposures, and contextual factors presented by these 2 populations (region, climate, seasonality, diet, etc.) provides a unique juxtaposition of divergent exposure settings. Additionally, since both studies used the same gold-standard analytical method, in the same laboratory, within a short time span, comparison of aflatoxin exposures—and in future work, the influence of aflatoxin on growth outcomes—can be conducted across these populations without concerns over methodological variance or harmonization. Moreover, the repeated-measures design of both trials allows for longitudinal assessment of aflatoxin exposure within individuals and links maternal and child exposures within a dyad over time.

There are limitations to our study. While the dietary source of AFB_1_ contamination is clear in Malawi (maize), we have not identified a primary source of exposure in Bangladesh. Additional work will be necessary to identify the source(s) of exposure in the Bangladeshi diet, as well as reveal low-risk foodstuffs that may be responsible for seasonally replacing contaminated dietary staples, leading to the annual and precipitous decline in AFB_1_ exposure that we observed in this population. Second, while a unified analytical platform allows side-by-side quantitative comparison of aflatoxin exposure, many demographic variables in these two populations were not measured in both settings, or in the same manner. Additionally, the timing and type of sample collection differed by population, leaving some comparative gaps that cannot be filled or that must be approached with caution (e.g., cord blood measurements allow direct assessment of *in utero* exposure in Bangladesh but were not available in Malawi). Subgroup sample sizes within and between studies were unequal, and neither cohort was recruited as a representative sample of the population as a whole; both of these factors could present challenges when attempting to generalize our findings.

In summary, we report a large, detailed survey of longitudinal aflatoxin exposure across a reproductive event in mother–child pairs enrolled in two randomized nutrition intervention trials, conducted in substantially differing aflatoxin exposure contexts on two continents. Aflatoxin exposure was prevalent throughout the year and of high magnitude in Malawi, although exclusive breastfeeding may temporarily mitigate nearly 60% of exposure in infants. In Bangladesh, exposure magnitude was lower, but substantial, and subject to significant seasonal variability; censored regression analysis and dosimetry calculations suggest that, after peak exposure at the end of the rainy season, Bangladeshi mothers eliminate 94% of their dietary AFB_1_ exposure every winter. These results suggest that exposure mitigation or primary prevention in both settings is possible, although potentially through different strategies. Our findings not only provide scientists, interventionists, and policymakers with a detailed examination of aflatoxin exposure in two at-risk populations but lay the foundation for follow-up studies examining the role of aflatoxin in child growth and development. Preventing aflatoxin exposure in the world's most vulnerable communities will become an even more pressing issue in the coming decades, as climate change is expected to substantially increase aflatoxin contamination of staple crops (76).

## Supplementary Material

nzab153_Supplemental_File

## Data Availability

Data utilized to generate this manuscript will be made available via repositories at Johns Hopkins University and the University of California-Davis for the JiVitA-3 and iLiNS-DYAD-M studies, respectively, and will be shared upon reasonable request.

## Notes

This work was supported by the Bill & Melinda Gates Foundation (BMGF) OPP1164205. The JiVitA-3 trial was funded by BMGF OPP614 (to Johns Hopkins University), with additional assistance for the trial provided by the Sight and Life Research Institute. The iLiNS-DYAD trial was funded by BMGF OPP49817 (to the University of California-Davis).

Author disclosures: The authors report no conflicts of interest. The Bill & Melinda Gates Foundation had no role in the design of the study, the collection, management, analysis, and interpretation of the data, or the preparation, review, and approval of the manuscript. In accordance with the grant conditions of the BMGF, a Creative Commons Attribution 4.0 Generic License has already been assigned to the Author Accepted Manuscript version of this submission.

Supplemental Figures 1–5 are available from the “Supplementary data” link in the online posting of the article and from the same link in the online table of contents at https://academic.oup.com/cdn/.

Abbreviations used: AFB_1_, aflatoxin B_1_; AFB_1_-lysine, AFB_1_-lysine albumin adduct; AFM_1_, aflatoxin M_1_; C-6mo, child at 6 mo of age; C-18mo, child at 18 mo of age; C-24mo, child at 24 mo of age; Cord, cord blood; FAST, Food Access Survey Tool; FWHM, full width at half-maximum; HAZ, height-for-age *z* score; HFIAS, Household Food Insecurity Access Scale; IDMS, isotope-dilution mass spectrometry; IFA, iron and folic acid; LAZ, length-for-age *z* score; LNS, lipid-based nutritional supplement; LOD, limit of detection; LOWESS, locally weighted scatterplot smoothing; M-1TM, maternal first trimester; M-2TM, maternal second trimester; M-3TM, maternal third trimester; M-3mo, maternal 3 mo postpartum; M-6mo, maternal 6 mo postpartum; MMS, multiple micronutrient supplement; n.d., not detected; QC, quality control; QC_0_, blank quality control sample; QC_L_, low quality control sample; QC_M_, medium quality control sample; QC_H_, high quality control sample; UHPLC-MS/MS, ultra-high-performance liquid chromatography–tandem mass spectrometry; WAZ, weight-for-age *z* score; WLZ, weight-for-length *z* score.
