# Supplementary material for: Longitudinal Assessment of Prenatal, Perinatal, and Early-Life Aflatoxin B1 Exposure in 828 Mother–Child Dyads from Bangladesh and Malawi
Source: Curr Dev Nutr. 2022 Jan 7;6(2):nzab153. doi: 10.1093/cdn/nzab153 (PMC8829025; doi:10.1093/cdn/nzab153)
Supplement: nzab153_Supplemental_File [file nzab153_supplemental_file.pdf]

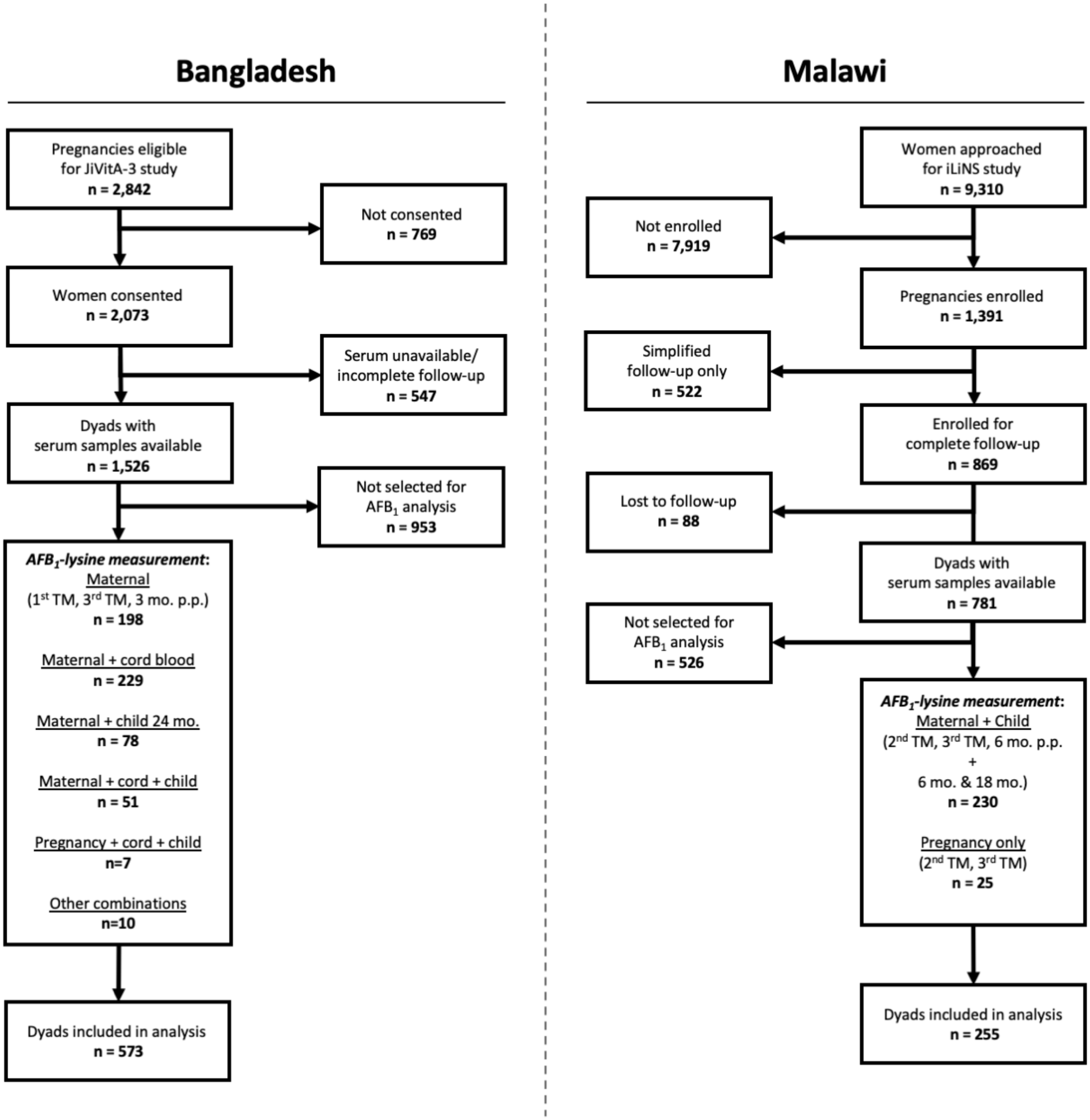

Supplemental Figure 1. CONSORT-style flow diagram summarizing selection and analysis of samples from the JiViTA-3 (Bangladesh) and iLiNS-DYAD-M (Malawi) trials for inclusion in the current study. All pregnancies included in the analysis resulted in singleton live births, with the exception of one pregnancy in Malawi (twins).

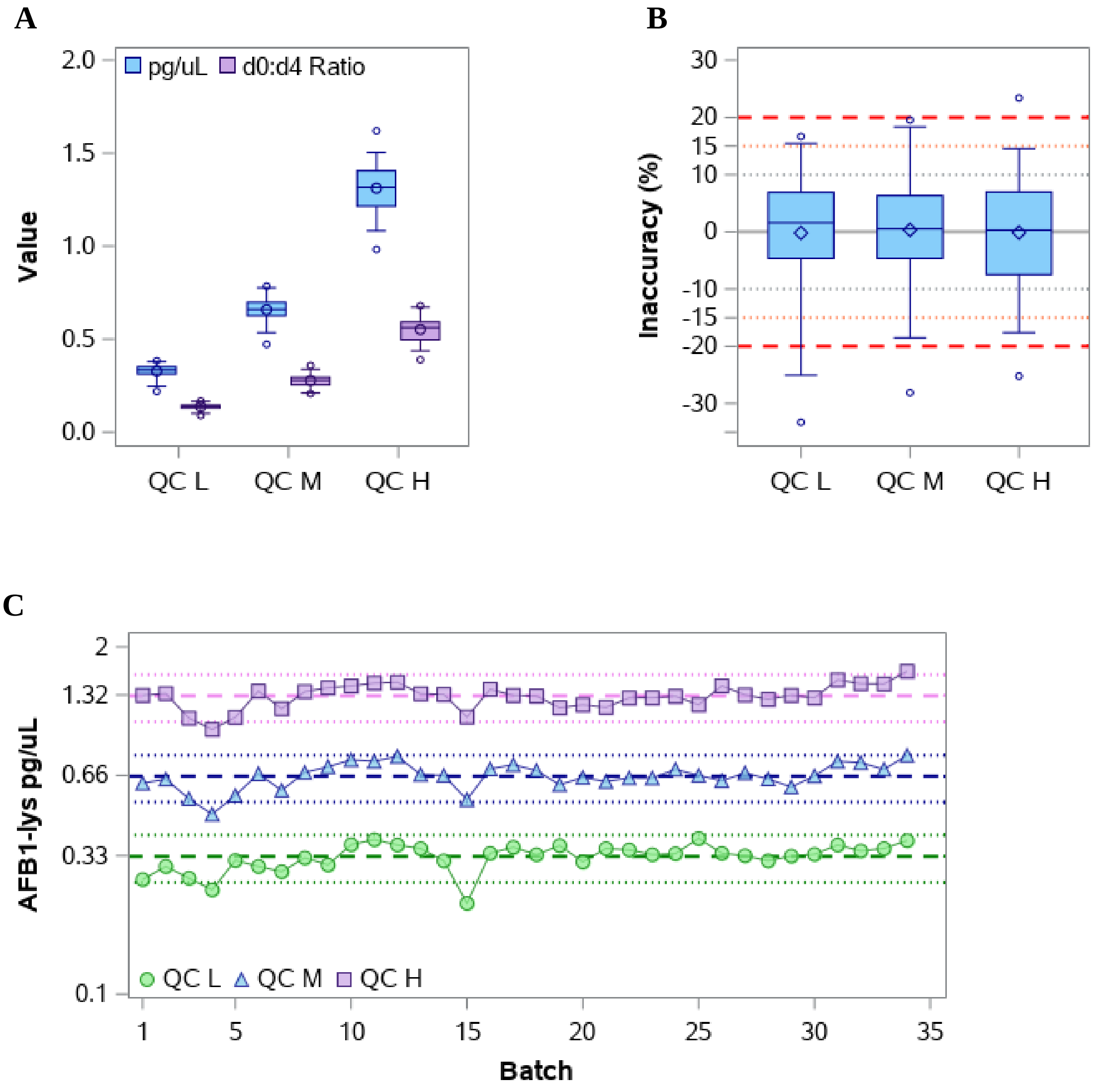

Supplemental Figure 2. Quantitative accuracy stable across ~4,500 injections. **A**) Box plot of the calculated concentrations and peak area ratios of unlabeled (d0) : isotopically labelled (d4) AFB_1-lysine for QC_L, QC_M, and QC_H quality control samples, prepared by diluting AFB_1-dosed rat serum into control human serum. **B)** Boxplot of quantitation inaccuracies for individual QC injections, relative to the consensus mean rat serum AFB_1-lysine concentration, which was calculated from all QC injections. **C)** Longitudinal stability of AFB_1-lysine quantitation across 34 batches with valid data. Dashed lines indicate the theoretical AFB_1-lysine concentration for each QC level; dotted lines indicate ±20% limits.

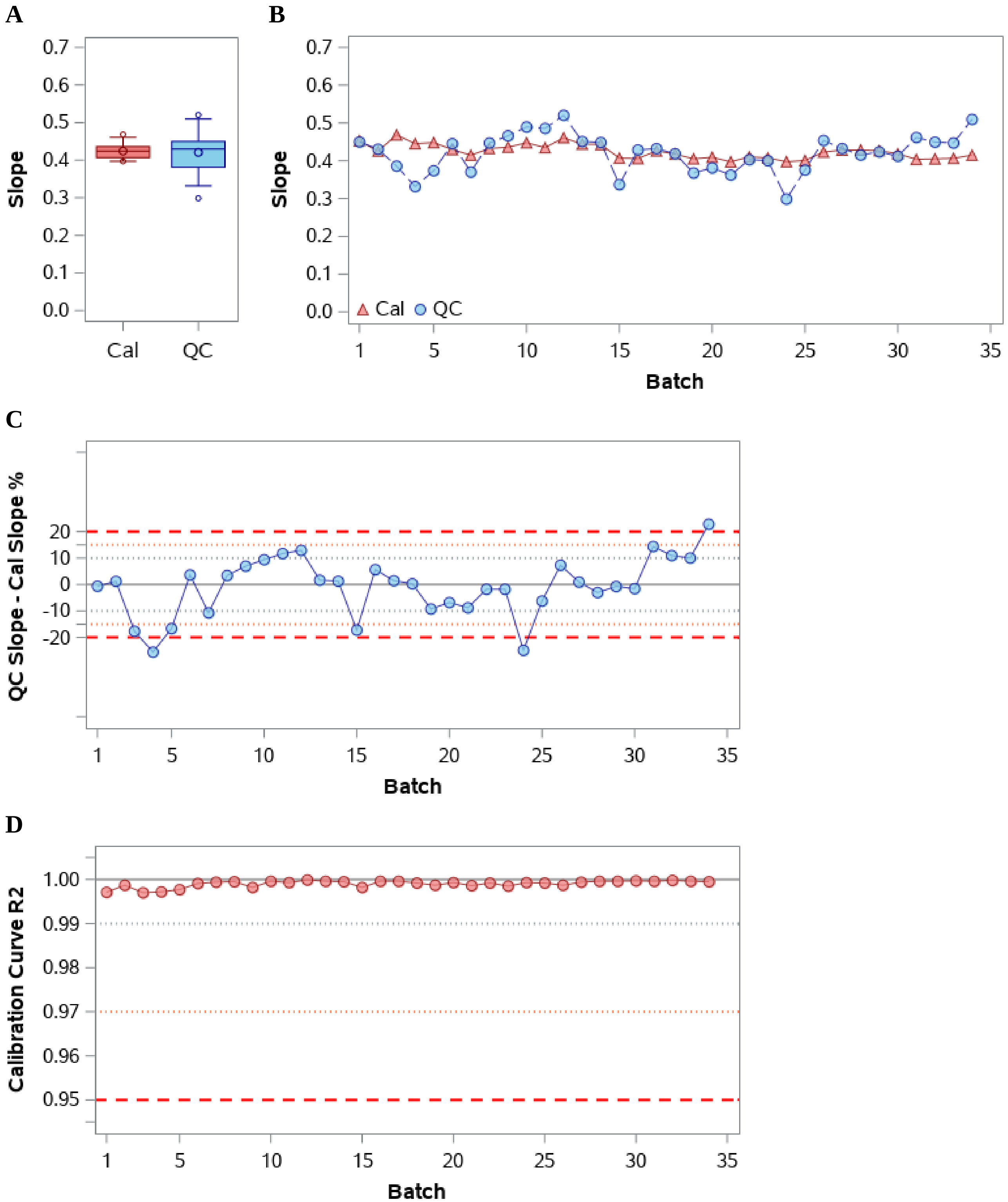

Supplemental Figure 3. Parallelism and stability of calibration curve across ~4,500 injections. **A)** Box plot of linear slopes for AFB_1-lysine calibration curves (in solvent) and QC samples (in serum matrix), across all assay

batches. **B,C)** Longitudinal concordance of calibrator and QC slopes. **D)** Longitudinal performance of calibration curve linear fit (R^2).

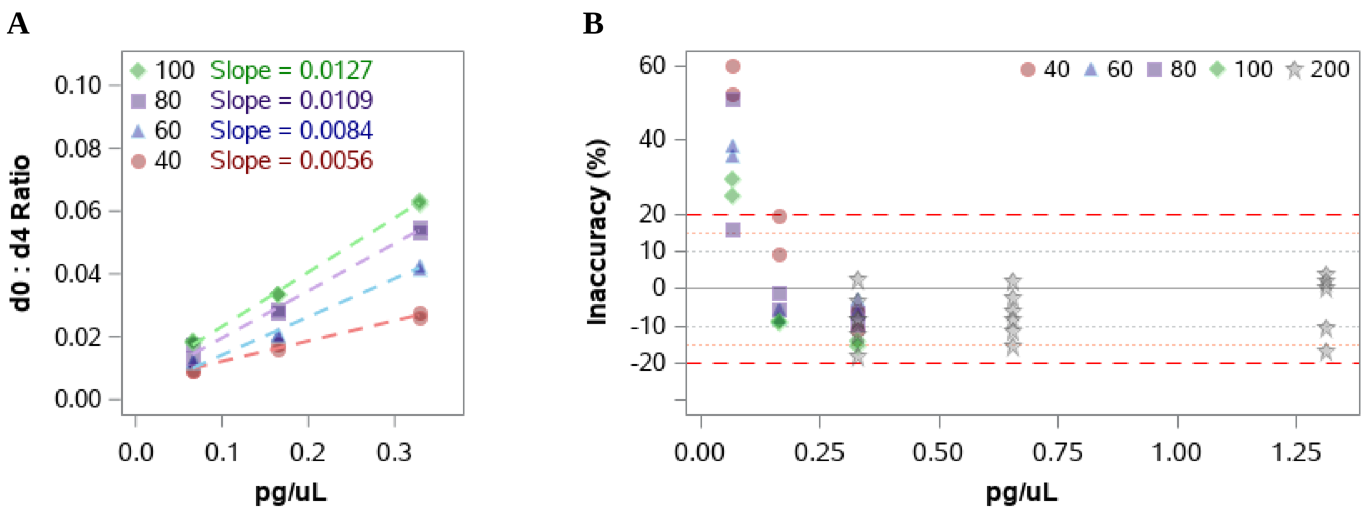

Supplemental Figure 4. Linearity of assay using low serum input volumes. **A**) Linearity of assay response at low AFB_1-lysine concentration and low input serum volume (n=2/condition). QC_L samples (100 µL, 80, µL, 60 µL, or 40 µL) were prepared without dilution (0.33 pg/µL) or diluted 2- or 4-fold in human control serum (0.17 pg/µL or 0.083 pg/µL). Samples were digested using the same protocol as for 200 µL input, but with PBS (1X, pH 7.2) replacing the omitted serum volume (*e.g.*, 100 µL serum + 100 µL PBS; 80 µL serum + 120 µL PBS, etc). Samples were subsequently processed and analyzed as usual. Data show a linear response for all levels of serum input, with decrements in slope proportional to reductions in serum input volume. **B)** Data represent the percentage difference between observed and expected AFB_1-lysine concentrations. Quantitative accuracy of assay is acceptable at concentrations > 0.150 pg/µL even with very low input volumes (40 µL), but decreases with combined low AFB_1-lysine concentration (< 0.08 pg/µL) and low input volumes (≤ 80 µL).

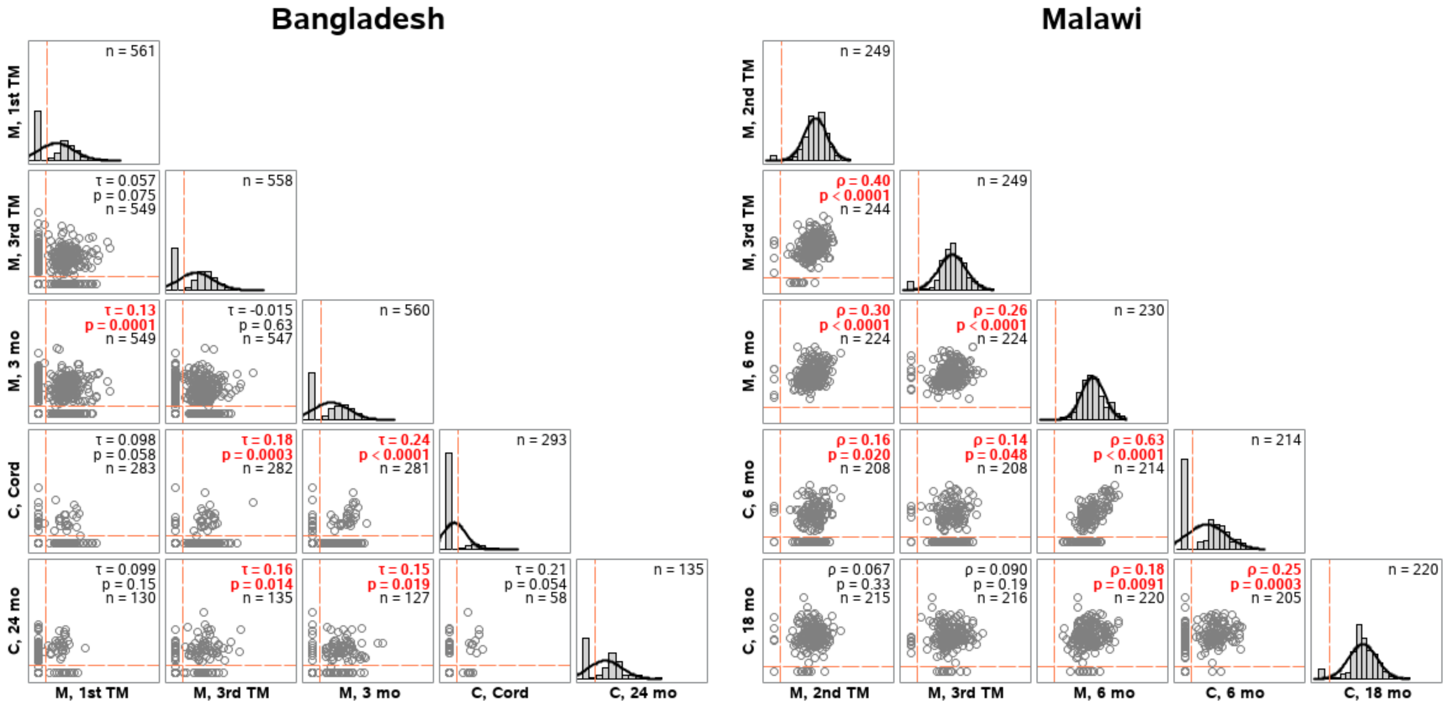

Supplemental Figure 5. Bivariate correlation matrices of AFB_1-lysine adduct levels in Bangladeshi and Malawian mothers and children. Scatter plots display $\log_{10}$-transformed AFB_1-lysine adduct concentrations (pg/µL). Diagonal displays histograms, normal distribution probability density functions, and sample sizes for each subgroup. Vertical and horizontal dashed lines within each plot indicate limit of detection (0.01 pg/µL); non-detectable values are imputed at 0.005 pg/µL. Values in red indicate statistically significant ($\alpha = 0.05$) Kendall's tau-b (*τ;* Bangladesh) or Spearman rank-order (*ρ;* Malawi) correlation coefficients. Axis values are omitted for clarity.
